# Molecular Evidence for Relaxed Selection on the Enamel Genes of Toothed Whales (Odontoceti) with Degenerative Enamel Phenotypes

**DOI:** 10.3390/genes15020228

**Published:** 2024-02-10

**Authors:** Jason G. Randall, John Gatesy, Michael R. McGowen, Mark S. Springer

**Affiliations:** 1 Department of Evolution, Ecology, and Organismal Biology, University of California, Riverside, CA 92521, USA; jrand006@ucr.edu; 2 Division of Vertebrate Zoology, American Museum of Natural History, New York, NY 10024, USA; johngatesy2@gmail.com; 3 Department of Vertebrate Zoology, Smithsonian National Museum of Natural History, MRC 108, P.O. Box 37012, Washington, DC 20013, USA; mcgowenm@si.edu

**Keywords:** enamel, Odontoceti, pseudogenes, relaxed selection, teeth, toothed whales

## Abstract

Different species of toothed whales (Odontoceti) exhibit a variety of tooth forms and enamel types. Some odontocetes have highly prismatic enamel with Hunter-Schreger bands, whereas enamel is vestigial or entirely lacking in other species. Different tooth forms and enamel types are associated with alternate feeding strategies that range from biting and grasping prey with teeth in most oceanic and river dolphins to the suction feeding of softer prey items without the use of teeth in many beaked whales. At the molecular level, previous studies have documented inactivating mutations in the enamel-specific genes of some odontocete species that lack complex enamel. At a broader scale, however, it is unclear whether enamel complexity across the full diversity of extant Odontoceti correlates with the relative strength of purifying selection on enamel-specific genes. Here, we employ sequence alignments for seven enamel-specific genes (*ACP4*, *AMBN*, *AMELX*, *AMTN, ENAM*, *KLK4*, *MMP20*) in 62 odontocete species that are representative of all extant families. The sequences for 33 odontocete species were obtained from databases, and sequences for the remaining 29 species were newly generated for this study. We screened these alignments for inactivating mutations (e.g., frameshift indels) and provide a comprehensive catalog of these mutations in species with one or more inactivated enamel genes. Inactivating mutations are rare in Delphinidae (oceanic dolphins) and Platanistidae/Inioidea (river dolphins) that have higher enamel complexity scores. By contrast, mutations are much more numerous in clades such as Monodontidae (narwhal, beluga), Ziphiidae (beaked whales), Physeteroidea (sperm whales), and Phocoenidae (porpoises) that are characterized by simpler enamel or even enamelless teeth. Further, several higher-level taxa (e.g., *Hyperoodon*, Kogiidae, Monodontidae) possess shared inactivating mutations in one or more enamel genes, which suggests loss of function of these genes in the common ancestor of each clade. We also performed selection (dN/dS) analyses on a concatenation of these genes and used linear regression and Spearman’s rank-order correlation to test for correlations between enamel complexity and two different measures of selection intensity (# of inactivating mutations per million years, dN/dS values). Selection analyses revealed that relaxed purifying selection is especially prominent in physeteroids, monodontids, and phocoenids. Linear regressions and correlation analyses revealed a strong negative correlation between selective pressure (dN/dS values) and enamel complexity. Stronger purifying selection (low dN/dS) is found on branches with more complex enamel and weaker purifying selection (higher dN/dS) occurs on branches with less complex enamel or enamelless teeth. As odontocetes diversified into a variety of feeding modes, in particular, the suction capture of prey, a reduced reliance on the dentition for prey capture resulted in the relaxed selection of genes that are critical to enamel development.

## 1. Introduction

Cetacea is the taxonomic group that includes whales, dolphins, and porpoises, and the crown clade is separated into two reciprocally monophyletic groups, one that includes the massive baleen-bearing filter feeders (Mysticeti), and the other that contains a variety of toothed forms (Odontoceti). Odontocetes display a range of tooth phenotypes that are associated with an array of feeding strategies and other behavioral traits. Some of the most striking differences are evident in comparisons of the polydont, enamel-covered teeth of raptorial dolphins (Delphinidae) and river dolphins (Inioidea (Iniidae + Pontoporiidae), Lipotidae, Platanistidae) versus the teeth of dwarf and pygmy sperm whales (*Kogia*) that are covered by thin prismless (“aprismatic”) enamel and the functionally toothless condition that is found in many suction-feeding female beaked whales (Ziphiidae) [1,2,3,4].

**Figure 1:**
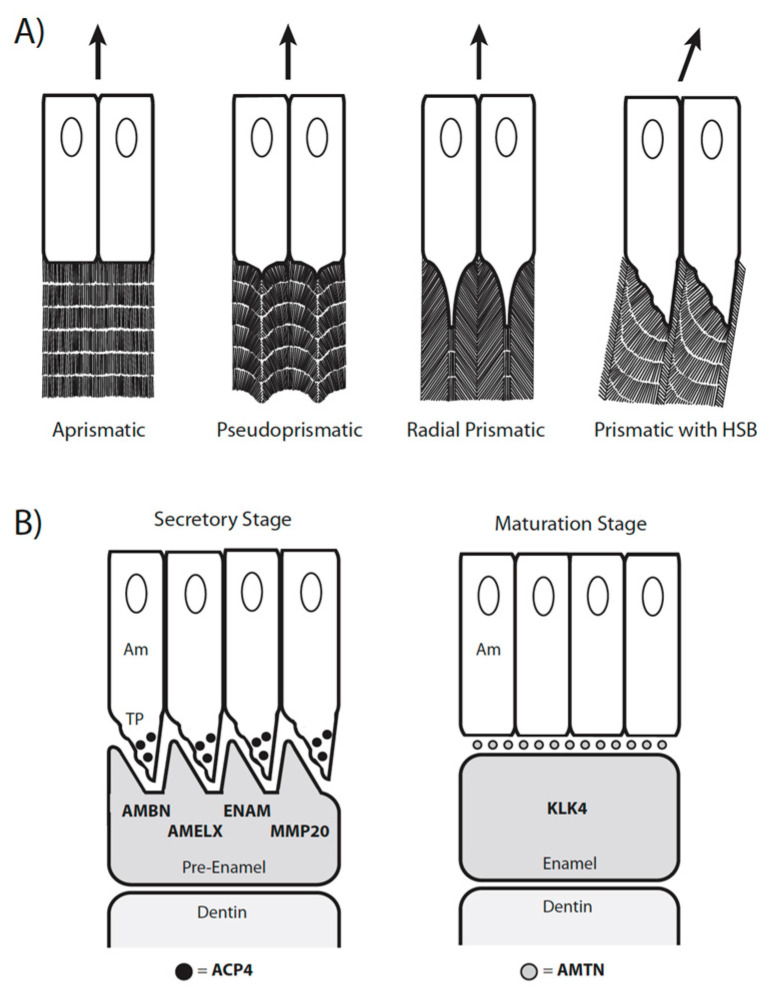
(**A**) Four types of enamel (aprismatic, pseudoprismatic, radial prismatic, prismatic with HSBs) that roughly correspond to Werth enamel complexity categories (2, 3, 4, 5, respectively). Arrows show the direction of ameloblast movement during amelogenesis. Aprismatic (=prismless) enamel is produced by ameloblasts that lack a Tomes’ process. Pseudoprismatic enamel is produced by ameloblasts that have a small Tomes’ process. Radial prismatic enamel without HSBs is produced by ameloblasts with a larger Tomes’ process and the enamel prisms follow a straight pathway from the dentin–enamel junction to the surface of the tooth crown. Prismatic enamel with Hunter-Schreger bands (HSBs) is produced by ameloblasts that follow a wavy pathway. Arrows show the direction of ameloblast migration. Figure modified from [8]. (**B**) Simplified overview of the secretory and maturation stages of amelogenesis to illustrate the expression of seven enamel genes that are important in enamel formation. In the secretory stage of amelogenesis, the enamel matrix proteins (EMPs) AMBN, AMELX, and ENAM are secreted by the Tomes’s processes (TP) of ameloblasts (Am) [10]. These proteins help to direct the organized deposition of hydroxyapatite crystallites into prisms and interprisms. ACP4 (black circles) processes and regulates the EMPs and is primarily localized in Tomes’s processes [11]. MMP20 cleaves the EMPs to produce functional peptides [10]. In the maturation stage of amelogenesis, AMTN (gray circles) is localized at the interface between the apical end of the ameloblast and the enamel surface [12]. AMTN plays an important role in hydroxyapatite nucleation. KLK4 degrades the EMPs, which are replaced by hydroxyapatite [10]. Protein abbreviations are in bold text.

There is also extensive variation in the complexity of the enamel microstructure in different odontocete species. Enamel complexity ranges from highly prismatic enamel with Hunter-Schreger bands (HSBs) in some river dolphins (*Inia geoffrensis, Platanista* spp.) to thin prismless enamel in pygmy and dwarf sperm whales (*Kogia* spp.) or no enamel at all in the narwhal (*Monodon monoceros*) [4,5,6]. Enamel, the outer covering of teeth, is highly mineralized and is composed primarily of hydroxyapatite crystals that are deposited in an orderly arrangement to form rods and interrods [7]. The development of these crystals is directed by enamel matrix proteins (EMPs) that are secreted by ameloblasts. The morphology of these enamel-forming cells varies greatly across different species [7,8]. Prismatic enamel, which is found in most mammals, consists of bundles of crystals (rod-like prisms) that are separated from each other by discontinuities (interprisms). Enamel prisms can follow a straight course from the dentin–enamel junction (DEJ) to the tooth surface (“radial prismatic enamel”) or exhibit a more complex pattern when different layers of ameloblasts follow wavy migration pathways in different directions from the DEJ to the tooth crown surface (Figure 1A). This orchestrated movement of ameloblasts results in prismatic enamel with HSBs [8]. This type of enamel contains adjacent layers of prisms that interweave with each other (i.e., decussate) at angles of up to ~90 degrees [9]. HSBs increase the strength of enamel and make it more resistant to fracture because the decussating layers of enamel resist crack propagation [8,9]. Prismatic enamel with HSBs is a key feature in the evolution of the dentition in many mammalian clades [8]. Conversely, the enamel of amphibians and most reptiles is prismless, and forms from ameloblasts that secrete EMPs from a flat surface (Figure 1A). Aprismatic enamel lacks the added strength that is found in radial prismatic enamel and especially in prismatic enamel with HSBs [8].

Crown cetaceans (Neoceti) evolved from archaeocete ancestors with highly prismatic enamel. Indeed, some archaeocetes (e.g., *Saghacetus osiris*, *Basilosaurus isis*) possessed prismatic enamel with HSBs [4]. Aside from oceanic dolphins (Delphinidae) and river dolphins (Inioidea, Lipotidae, Platanistidae), which retain the most complex enamel among extant odontocetes, living representatives of other families of toothed whales that have been investigated have more simple or degenerative enamel with no HSBs [4,5,6,13]. Simplified enamel phenotypes may have arisen in conjunction with changes in feeding strategy, changes in other behaviors, and/or new interactions between teeth and their surrounding aquatic environment. Among mammals, in addition to prey acquisition and food processing, teeth are also used for defense, male–male competition, and sensing external stimuli [4,14,15,16]. However, some of these uses for teeth may be unnecessary for most cetaceans. Cetaceans do not have many predators due to their large size, are generally not territorial (but see [17]), and do not need to protect breeding ranges [18,19]. Many extant odontocetes feed by grasping prey items with their teeth and then swallowing prey whole instead of chewing and then swallowing masticated food items as in most mammals (some exceptions include odontocete species such as the killer whale *Orcinus orca* and the Amazon River dolphin *Inia geoffrensis*) [15,20]. Other odontocetes, including the narwhal and beaked whales, are suction feeders and primarily use their teeth for display or male–male competition [4,21]. It has even been suggested that some odontocetes such as sperm whales and bottlenose dolphins may use high intensity sounds to debilitate prey prior to ingestion, thereby reducing the importance of strong teeth for prey capture [21,22].

Werth et al. [4] assessed whether enamel complexity is correlated with different ecological parameters, life history variables, and/or the extent to which upper and lower teeth occlude with each other. To perform their correlation analyses, Werth et al. [4] assigned discrete enamel organization scores to each taxon that was included in their study as follows: 1 = no enamel, 2 = prismless enamel, 3 = intermediate or irregular enamel that corresponds to Ishiyama’s [5] pseudoprismatic enamel, 4 = prismatic enamel, and 5 = enamel with HSBs or other decussation (Table 1 in [4]). Importantly, each increase or decrease in enamel complexity corresponds to the addition or subtraction of a fundamental biological feature of enamel. These authors concluded that less complex enamel types (lower rankings) are correlated with feeding on softer prey with less oral processing, possessing fewer teeth, and having wider jaws [4]. Within Odontoceti, reduced mastication and mechanical demands on teeth therefore may have initiated relaxed purifying selection on the enamel structure and the underlying enamel-related genes [6].

Some of the genes that are involved in enamel formation include amelogenin (*AMELX*), ameloblastin (*AMBN*), enamelin (*ENAM*), and acid phosphatase 4 (*ACP4*), all of which are strongly expressed in the secretory stage of amelogenesis (Figure 1B) [10,23,24]. Of these, *AMELX*, *AMBN*, and *ENAM* encode EMPs that are cleaved by matrix metallopeptidase 20 (MMP20) during the secretory stage to produce functional peptides, and are ultimately degraded by kallikrein-related peptidase 4 (KLK4) prior to their removal by endocytosis and replacement with hydroxyapatite crystallites during the maturation stage of amelogenesis (Figure 1B) [7,10,23,25,26]. Amelotin (*AMTN*) and odontogenic ameloblast-associated (*ODAM*), in turn, encode proteins that are expressed during the maturation stage of amelogenesis and have a role in hydroxyapatite nucleation [12,25]. The genes for two of the EMPs (*AMBN*, *ENAM*), *AMTN*, and *ODAM* are tightly linked on the same chromosome in the secretory calcium-binding phosphoprotein (SCPP) gene cluster, which also includes genes for dentin/bone calcification, milk production, and antimicrobial activity in the saliva; *AMELX* is also an SCPP gene but is located on the X-chromosome [27,28,29].

Due to the loss of teeth in mysticetes as well as the peculiarities of enamel in various odontocetes, tooth genes have been screened for inactivating mutations in Cetacea. Springer et al. [30] reported that the tooth-related gene *ODAM* is inactivated in all the odontocetes that were examined (eight species). *ODAM* loss may be related to the simplified enamel structure in these whales and/or the altered antimicrobial functions of the junctional epithelium necessary for aquatic environments [30].

Studies investigating enamel-specific genes (*ACP4*, *AMBN*, *AMELX*, *AMTN*, *ENAM*, *KLK4*, *MMP20*) have reported a variety of inactivating mutations in Odontoceti. Most of these mutations were reported in species that have the least complex enamel such as *Mesoplodon bidens* (Sowerby’s beaked whale), *Monodon monoceros* (narwhal), *Physeter macrocephalus* (sperm whale), *Kogia* spp. (pygmy and dwarf sperm whales), and *Delphinapterus leucas* (beluga) [31,32,33,34,35]. However, mutations have also been reported in taxa with more complex enamel such as porpoises (Phocoenidae) with intermediate enamel and the killer whale (*Orcinus orca*) that has prismatic enamel [33,34,35]. At the same time, enamel-specific genes have only been examined in a small sample of odontocete species.

In addition to reporting inactivating mutations, Randall et al. [35] performed branch-specific selection analyses on enamel genes in odontocetes and observed elevated dN/dS values on the terminal branches for *Mesoplodon bidens* and *Physeter macrocephalus*, and on the stem and crown branches for *Kogia*, Monodontidae, and Phocoenidae. These dN/dS values were higher than for outgroup taxa with functional enamel-capped teeth, and indicate relaxed purifying selection on enamel-specific genes in these lineages and clades.

Here, we build on Randall et al.’s [35] prior investigation of tooth genes in cetaceans and assemble sequence alignments for seven enamel-specific genes that are known to exhibit inactivating mutations in vertebrate taxa that either lack teeth or have enamelless teeth. Our expanded data set includes 62 odontocete species, 13 mysticete species, and 17 terrestrial and semiaquatic cetartiodactyl outgroup species, including new sequences for 29 odontocete species. Alignments were screened for inactivating mutations including frameshift insertions and deletions, premature stop codons, intron splice site mutations, start and stop codon mutations, and deletions of entire exons or genes. We performed ancestral reconstructions with enamel complexity scores from Werth et al. [4] and calculated the number of inactivating mutations per million years (MYRs) on each branch of the odontocete tree. We also performed selection analyses to assess the extent to which purifying selection has been relaxed in odontocete clades with simpler versus more complex enamel. Finally, we used Spearman’s rank-order correlation and linear regression to test the hypothesis that purifying selection has been relaxed more on branches with simple enamel or no enamel relative to branches with higher enamel complexity. The results of this study provide the first comprehensive phylogenetic assessment of a correlation between enamel complexity and selection intensity on enamel-specific genes in Cetacea.

## 2. Materials and Methods

### 2.1. Gene Sampling

Genes included in this study (*ACP4*, *AMBN*, *AMELX*, *AMTN*, *ENAM*, *KLK4*, *MMP20*) are shown in Figure 1B and were chosen based on (1) prior studies that reported inactivation of these genes in edentulous (turtles, birds, pangolins, baleen whales, anteaters, Steller’s sea cow) and enamelless vertebrates (aardvark, armadillo, sloth), (2) mutations in humans that cause amelogenesis imperfecta, and (3) mutagenesis gene knockout studies in mice [10,24,31,32,33,34,35,36,37,38,39,40,41,42,43,44]. The tooth-related gene *ODAM* was also pseudogenized in all odontocete species that were examined by Springer et al. [30]. However, this gene is inactivated in several other mammalian clades with enamel-capped teeth and was omitted from the present study. Exon 4 of *AMELX* was excluded because this exon is subject to alternative splicing and is absent in many mammals [45,46,47,48].

### 2.2. Taxon Sampling

Taxon sampling included 92 species of which 62 are odontocetes, 13 are mysticetes, and 17 are terrestrial or semiaquatic cetartiodactyl outgroups (Table 1). Odontocete and mysticete sampling included all species for which genome data are available. Outgroup sampling included a diverse assortment of taxa that are representative of the major terrestrial and semiaquatic clades (Tylopoda, Suina, Ruminantia, Hippopotamidae).

In addition to the full data set with 92 species and two subspecies of *Delphinus delphis*, we performed a subset of analyses on a pruned data set that contained 37 taxa (33 odontocetes and 4 cetartiodactyl outgroups). The 33 odontocetes in the pruned data set comprise species with molecular data for enamel-specific genes plus scores for Werth et al.’s [4] index of enamel complexity. This data set includes at least one species from each genus and each family in Werth et al.’s [4] original data set (Table 2, also see below).

### 2.3. BLAST Searches and Procurement of Molecular Data

DNA sequences for seven different tooth genes (*ACP4*, *AMBN*, *AMELX*, *AMTN*, *ENAM*, *KLK4*, *MMP20*) were obtained from (1) the assembled genomes at NCBI (https://www.ncbi.nlm.nih.gov/, accessed on 6 February 2024), DNA Zoo (https://www.dnazoo.org/, accessed during calendar years of 2021, 2022, and 2023) [49], and The Bowhead Whale Genome Resource (http://www.bowhead-whale.org/, accessed during calendar years of 2021, 2022, and 2023) [50], (2) raw sequence reads at NCBI’s Sequence Read Archive (SRA), and (3) newly generated Illumina whole-genome sequence data (JG, MRR, MSS) (see Table S1 for accession numbers and/or sources for each species). NCBI’s RefSeq and Nucleotide databases were searched using keywords for all seven genes in conjunction with taxon names for four reference species (*Capra hircus*, *Camelus bactrianus*, *Orcinus orca*, *Tursiops truncatus*). The sequences for each reference species were then imported into Geneious Prime (version 2022.2.2, https://geneious.com, accessed on 6 February 2024) [51], aligned with MAFFT [52], and cross-checked against each other for consistent annotations. Sequences for additional species were procured using NCBI’s Nucleotide Basic Local Alignment Search Tool (BLAST), which was used to search both assembled and unassembled genomes using the whole-genome shotgun (WGS) and SRA databases, respectively. Each BLAST search employed a query sequence from a closely related species. MegaBLAST was used for highly similar sequences (e.g., taxa in same family) and Blastn was used for less similar sequences (e.g., taxa in different families). Top-scoring BLAST results were imported into Geneious Prime. Sequences obtained through the SRA database were assembled using Geneious Prime’s ‘Map to Reference’ approach, where the reference sequence was a closely related species to the SRA taxon. We allowed for a maximum mismatch of 10% per read and required a minimum of two reads for base calling with a consensus threshold of 65%. We also used the map to reference approach in Geneious Prime to assemble genes of interest for 30 odontocete specimens (Table S1) for which we generated paired-end Illumina sequence reads. DNA samples for these libraries were provided by the Southwest Fisheries Science Center and the Smithsonian National Museum of Natural History. We again allowed for a maximum mismatch of 10% per read with a closely related reference taxon and required a minimum of two reads for base calling with a consensus threshold of 65%. Most DNA libraries for the odontocete samples were constructed with Illumina’s NeoPrep procedure after sonicating the samples to a mean length of 550 bp at the University of California, Riverside (UCR), Genomics Core Facility. In the case of four *Mesoplodon* species (*M. bowdoini, M. ginkgodens, M. grayi, M. layardii*), libraries were constructed with a NEB Ultra II DNA kit with dual unique indexes. Finally, DNA libraries for four species (*Hyperoodon ampullatus, Lagenorhynchus albirostris, Mesoplodon mirus, Mesopolodon perrini*) were constructed using Illumina New England Biolabs NEBNext Ultra DNA Library Prep Kits at the Smithsonian National Museum of Natural History (NMNH) Laboratory of Analytical Biology (LAB). With the exception of the four *Mesoplodon* libraries that were prepared with the NEB Ultra II DNA kit, all libraries were then sequenced at ~30–45× coverage at the New York Genome Center using paired-end sequencing (150 bp per read) on a HiSeq 2500 platform. The four *Mesoplodon* libraries were sequenced at the same coverage on a NovaSeq platform (150 bp paired-end sequencing).

### 2.4. Alignments and Inactivating Mutations

Complete protein-coding sequences and introns were aligned in Geneious Prime using MAFFT [52]. Sequences were manually spot-checked for alignment errors using AliView version 1.28 [53]. Alignments were manually screened for different types of inactivating mutations in exons and introns that are regularly inferred from genome sequences including frameshift insertions and deletions (indels), start and stop codon mutations, premature stop codons, intron splice site mutations, exon deletions, and whole gene deletions [31,35,43]. All of these mutations were annotated in Geneious Prime. Synapomorphic inactivating mutations were validated by their occurrence in two or more taxa. Autapomorphic inactivating mutations in odontocete taxa for which we generated Illumina data were validated by their occurrence in multiple Illumina reads. In the case of autapomorphic inactivating mutations that were detected in the genome sequence of a single taxon, we BLASTed (MegaBLAST) genomic segments containing these mutations to the associated Illumina reads on NCBI’s Sequence Read Archive (Table S2). Mutations with allelic variation (see Table 3) were each counted as 0.5 mutations when the two alternatives were an inactivating mutation versus the ancestral (presumed functional) condition. These were determined by verifying heterozygous mutations in alignments of mapped sequence reads, with criteria of at least 10× coverage and each allele consisting of at least 40% of the reads (Supplementary Table S2).

Inactivating mutations were mapped onto species trees (see Section 2.7) with delayed transformation (DELTRAN) character optimization. Mapping with accelerated transformation (ACCTRAN) character optimization yielded identical results. DELTRAN mapping was performed on the full data set for 93 taxa and the pruned data set for 37 taxa. For the latter, the number of inactivating mutations on each branch was divided by the length of the branch in millions of years on the corresponding species timetree (see Section 2.7). This resulted in mutations/MYRs for each branch and these values were used in correlation and linear regression analyses to test for an association between the number of inactivating mutations/MYRs and enamel complexity (see Section 2.10).

### 2.5. Phylogenetic Analyses

Gene trees were constructed from the aligned protein-coding sequences with RAxML, which is a widely used maximum likelihood program for inferring gene trees and species trees, e.g., ref. [43]. We used RAxML version 8.2.11 in Geneious Prime (raxmlHPC-SSE3-MAC) [54]. Rapid bootstrapping (500 pseudoreplicates) and a search for the best tree were performed in the same analysis [55]. We employed the GTRGAMMA option, which implements the GTR + Γ model of DNA sequence evolution [56].

### 2.6. Collection of Data for Enamel Complexity

We compiled an enamel complexity data set (Werth enamel complexity, Table 2) for a subset of species from our full data set. Taxon sampling for this data set included 29 odontocetes with enamel complexity scores from Werth et al. (Table 1 in [4]) plus four additional odontocetes and four cetartiodactyl outgroups for which we scored enamel complexity based on the literature. The additional odontocetes were *Monodon monoceros*, *Neophocaena asiaeorientalis*, *Platanista minor*, and *Kogia sima*. The cetartiodactyl outgroups, in turn, were *Hippopotamus amphibius*, *Bos mutus*, *Sus scrofa*, and *Camelus bactrianus.* The enamel complexity categories used in this study were the same as in Werth et al. [4]: 1 = no enamel, 2 = prismless enamel, 3 = intermediate or irregular enamel, 4 = prismatic enamel, and 5 = enamel with HSBs or other decussation. For taxa that were scored by Werth et al. [4], we used the same scores, including taxa that were scored with polymorphic enamel categories based on indeterminate results or specimens that showed notable variation. For odontocetes that were not scored by Werth et al. [4], the narwhal (*M. monoceros*) was scored as category 1 because this taxon lacks enamel on both the erupted (left side) and unerupted (right side) tusks [5,57]. The narrow-ridged finless porpoise (*Neophocaena asiaeorientalis*) was assigned the same score (3) as the finless porpoise (*Neophocaena phocaenoides*), given the very close relationship of these taxa. *N. asiaorientalis* previously was considered a subspecies of *N. phocaenoides* [58], but has been elevated to a full species based on genomic data [59]. The Indus River dolphin (*Platanista minor*) was assigned a score of 5, which is the same as its sister-species, the Ganges River dolphin (*Platanista gangetica*) [4]. The Indus River dolphin was described as having well-developed enamel with undulating HSBs [60]. For *Kogia*, we scored the dwarf sperm whale (*K. sima*) as category 2, the same category given to its sister species *Kogia breviceps*. Different studies have reported that this species has lost the enamel on its teeth or has a thin layer of enamel that quickly wears away [61,62,63,64]. All outgroup taxa were scored as category 5 [65,66,67,68].

### 2.7. Species Trees

The 93-taxon species tree that was used for Coevol analyses and mapping inactivating mutations was based on McGowen et al. [69], with augmentations from McGowen et al. [70] (*Berardius arnuxii*, *Cephalorhynchus eutropia*, *Cephalorhynchus hectori*, *Hyperoodon planifrons*, *Phocoena sinus*, *Platanista minor*), Zurano et al. [71] (*Indopacetus pacificus*), and Chehida et al. [72] (*Neophocaena asiaeorientalis*) for odontocete species that were not included in McGowen et al. [69]. Relationships among cetartiodactyl outgroups were based on Foley et al. [73]. We pruned the 93-taxon tree to 37 taxa for codeml analyses and ancestral reconstructions with the 37-taxon Werth enamel complexity data set.

Divergence times for the 37-taxon tree were based on McGowen et al.’s [69] Table S3 “Full dataset 6-partition AR (Mean)” except for several nodes that were not included in McGowen et al.’s [69] timetree. Specifically, divergence times for nodes 7, 42, and 63 were taken from McGowen et al. (mean dates from Table 2 in [70]), and the divergence time for node 52 was taken from Chehida et al. (Table S5 in [72]) (see Figure S1 for node numbers). The 93-taxon and 37-taxon species trees, with divergences times for the latter, are provided in the Supplementary Materials.

### 2.8. Ancestral Reconstructions of Enamel Complexity

Ancestral reconstructions for Werth enamel complexity were performed in PAUP* version 4.0a [74] and Mesquite version 3.70 [75]. PAUP was employed with DELTRAN, ACCTRAN, and most parsimonious reconstruction sets (MPR) settings. PAUP’s MPR setting and Mesquite both allow for multiple (equivocal) state assignments for a given node. In order to maintain a one-to-one correspondence between enamel complexity data and ancestral reconstructions, taxa that were scored as polymorphic for Werth enamel complexity (e.g., 1/2) were also scored as polymorphic in both PAUP and Mesquite ancestral reconstruction analyses. Enamel complexity was treated as an ordered character for all analyses in both programs. External and internal branches were assigned enamel complexity scores based on the enamel scores for the nodes on the basal and apical ends of each branch (Figures S2–S4). If the nodes at both ends of a branch had the same score for Werth enamel complexity, then the branch was assigned the same score (e.g., 3 → 3 = 3). If the nodes at the two ends of a branch had different scores, then the branch was assigned the mean value of these two scores (e.g., 4 → 2 = 3). External nodes with polymorphic character states and internal nodes that were reconstructed as equivocal (i.e., multiple states) were assigned mean values between the two states prior to calculating a value for the relevant branch (e.g., 1 → 1/2 = 1 → 1.5 = 1.25; 1/2 → 1/2 = 1.5 → 1.5 = 1.5).

### 2.9. Selection Analyses

Selection analyses (dN/dS) were conducted with two different programs, the codeml program of PAML version 4.9j [76] and Coevol version 1.6 [77]. Selection analyses were performed with a concatenation of seven enamel-specific genes (*ACP4*, *AMBN*, *AMELX*, *AMTN*, *ENAM*, *KLK4*, *MMP20*) that serve as a proxy for selection intensity on enamel. Importantly, analyses with the seven-gene concatenation are less impacted by sampling error than are analyses with individual genes. The analysis with Coevol included 93 taxa and 9627 base pairs (bp), whereas the analyses with codeml employed a reduced data set that included 37 taxa (i.e., Werth enamel complexity taxa) and 9525 bp. We used a rooted species tree for Coevol and an unrooted species tree for codeml as required by these programs (Section 2.7. Species Trees). All frameshift insertions were deleted for both Coevol and codeml analyses as is required for running these programs. Premature stop codons were recoded as missing data (NNN) prior to performing selection analyses with codeml [30,31]. Premature stop codons were automatically recoded as missing data by Coevol [78].

Selection analyses with codeml were performed with a free-ratio model wherein dN/dS values for each branch in the tree were estimated, and two different codon frequency models, CF1 and CF2 [76], were utilized. CF1 estimates codon frequencies from mean nucleotide frequencies across all three codon positions, whereas CF2 estimates frequencies at each of the three codon positions. Codon positions are absent in pseudogenes so it is important to verify that analyses without base compositional differences at different codon positions (i.e., CF1) yield results that are similar to results that are obtained with a codon frequency model that allows for base compositional differences at 1st, 2nd, and 3rd codon positions (i.e., CF2) [35,79].

By contrast with the maximum likelihood-based codeml, Coevol utilizes a Bayesian approach and provides a visual representation of dN/dS ratio estimates across different branches in a phylogeny [80]. The Coevol analysis was conducted on the 93-taxon species tree with 10 fossil calibrations obtained from McGowen et al.’s study [69] (Table 2). The age of the root node for this tree (65.83 Ma, Cetartiodactyla) was taken from McGowen et al.’s study [69] (Table S3), and was also used as the standard deviation for this analysis [78]. We employed the *dsom* procedure that uses a codon model with two a priori independent values (dS and dN/dS) as a priori independent variables. The data set was run for at least 200 cycles, and dN/dS values were sampled once every cycle. The burnin was determined with the *tracecomp* command, which was used to check for MCMC convergence by monitoring effective sample size.

### 2.10. Statistical Analyses

To test the significance of the relationships between Werth enamel complexity values and two different measures of selection at the molecular level (number of inactivating mutations/MYRs, dN/dS values), we analyzed our data set using Spearman’s rank-order correlation and a linear regression model. Correlation and regression analyses with the number of inactivating mutations/MYRs employed values for every internal and external branch of the rooted species tree. Analyses with dN/dS employed values from free-ratio selection analyses with the unrooted species tree. In the case of very short branches, codeml can return inexact dN/dS estimates that are either very low (e.g., 0.0001) or very high (e.g., 999.0). Inexact dN/dS estimates occur when there are no nonsynonymous and/or synonymous substitutions. To mitigate against this sample size problem, we omitted all branches with less than five substitutions. This resulted in 55 branches that were retained for the correlation and regression analyses with dN/dS values. Spearman’s rank-correlation analyses were performed with Wessa (https://www.wessa.net/stat.wasp, accessed on 6 February 2024). Linear regression analyses were performed with GraphPad (graphpad.com) and RStudio version 2023.06.2 Build 561 [81] using the libraries plyr, dplyr, ggplot2, and sjPlot with the linear regression function.We used MathCracker (https://mathcracker.com/, accessed on 6 February 2024) to calculate regression residuals and to perform normality tests (Anderson–Darling) on these residuals. For all of the regression analyses, molecular variables were selected as the independent variable and enamel complexity was selected as the dependent variable because enamel genes encode for proteins that produce enamel.

## 3. Results

### 3.1. Alignments and Gene Trees

Complete coding sequence alignments for all seven enamel genes are provided in the Supplementary Materials. Coding sequence alignments range from 645 bp (*AMTN*) to 3872 bp (*ENAM*). The number of sequences in the individual gene alignments ranges from 88 (*KLK4*) to 93 (*ACP4, AMELX, ENAM, MMP20*). Intact coding sequences were recovered for all of the terrestrial and semiaquatic cetartiodactyl outgroup taxa that were sampled, as well as for most of the odontocete species. Notable exceptions include the absence of *KLK4* in both species of *Kogia* and the absence of *AMBN* in *Kogia sima*. The sequences for three mysticetes are also missing for *KLK4* (Table S1).

Table S3 summarizes the presence or absence of 30 well-supported clades in Cetartiodactyla [69,70,82,83,84,85] for all seven gene trees. A majority of these well-supported clades were recovered by all seven gene trees with a mean of 25.43 clades per gene tree (~86%). *ENAM* has the longest coding sequence alignment among enamel-related genes (3522 bp), and the *ENAM* gene tree has the maximum number of recovered clades (30/30). *AMELX*, in turn, has the second shortest coding sequence alignment (693 bp), and the *AMELX* gene tree exhibits the fewest recovered clades (22/30).

### 3.2. Inactivating Mutations

**Figure 2:**
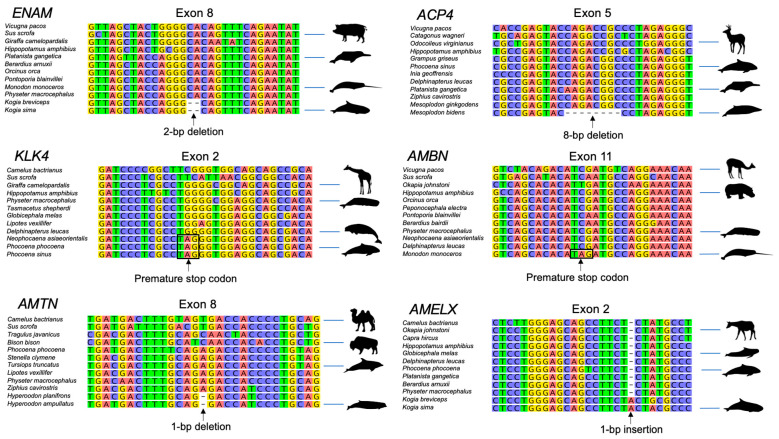
Examples of inactivating mutations in odontocete enamel genes.

**Figure 3:**
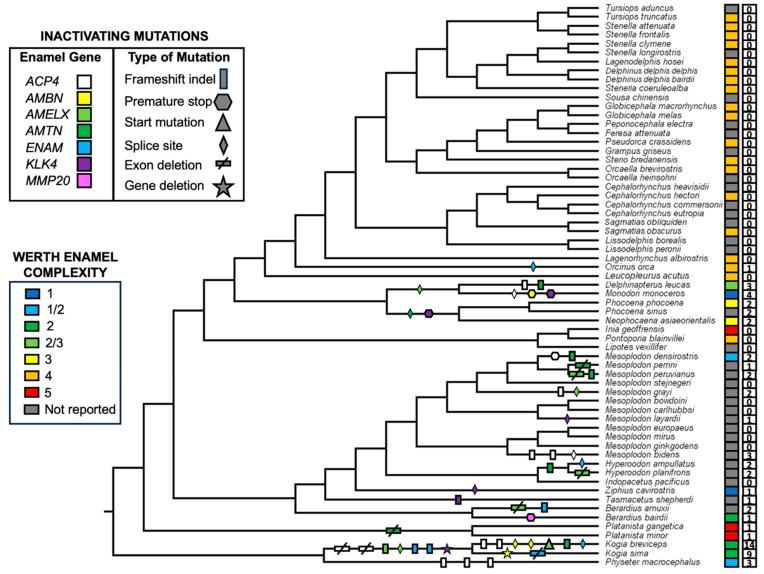
Mapping of inactivating mutations in seven enamel genes onto a species tree for 63 odontocetes with delayed transformation (DELTRAN) character optimization. Werth enamel complexity values are denoted to the right of taxon names with colored rectangular boxes. Open boxes with numbers indicate the number of inactivating mutations (synapomorphic + autapomorphic) in each taxon. Alignment coordinates of inactivating mutations are provided in Table 3. Accelerated transformation (ACCTRAN) character optimization resulted in identical mapping of inactivating mutations.

Coding sequences for all of the semiaquatic and terrestrial cetartiodactyl taxa that were sampled are intact. By contrast, all seven of the enamel genes exhibit at least one inactivating mutation in Odontoceti (Table 3). Examples of inactivating mutations are illustrated in Figure 2 and Figure S5. The number of inactivating mutations in different enamel genes ranges from two (*MMP20*) to fourteen (*ACP4*). Also, there are fewer mutations in taxa with more complex enamel phenotypes (Werth categories 4 and 5) and more mutations in taxa with less complex enamel phenotypes (Werth categories 1–3) (Figure 3, Table 3). Werth enamel complexity scores are 4 or 5 for all representatives of *Platanista*, Inioidea, and Delphinidae that have been scored for enamel complexity (Figure 3), and there are only two inactivating mutations (one synapomorphic, one autapomorphic) among 36 species that belong to these three clades. Of these, the only synapomorphic mutation is an in-frame deletion of exon 3 in the *AMTN* gene of the Ganges and Indus River dolphins (*Platanista*) that exhibit the most complex enamel phenotype (category 5). By contrast, Werth enamel complexity scores range from 1 to 3 for monodontids, phocoenids, ziphiids, and physeteroids that have been scored, and there are 46 inactivating mutations (11 synapomorphic, 35 autapomorphic) among 27 taxa that belong to these clades. For these taxa with simpler enamel, the most inclusive synapomorphic inactivating mutations are found in Monodontidae (*Monodon monoceros* + *Delphinapterus leucas*), Phocoenidae, *Kogia*, and *Hyperoodon* (Figure 2, Table 3). The longest inactivating mutation is a presumed whole gene deletion (*KLK4*) in *Kogia* based on the absence of BLAST hits and map to reference results. The two species of *Kogia* also share deletions of exons 1–3 and 11 in *ACP4* (Figure 2 and Figure S5, Table 3), frameshift indels in *ENAM*, and a frameshift indel and splice site mutation in *AMELX*. Both kogiids have autapomorphic mutations in *AMBN* and *K. breviceps* has a frameshift indel in *AMTN*. Hence, the only enamel gene with intact coding sequences in both species of *Kogia* is *MMP20*. There are no shared inactivating mutations for Ziphiidae, but 12 of the 19 beaked whales included in this study have at least one gene that contains an inactivating mutation (Figure 3, Table 3). These mutations are spread across six of the seven enamel genes and the only gene that is intact in all of the ziphiid species is *AMBN*. Shared inactivating mutations are also absent for Physeteroidea, although all three physeteroids exhibit inactivating mutations in at least one gene and *K. breviceps* exhibits mutations in six of the seven enamel genes as noted above.

### 3.3. Selection Analyses

**Figure 4:**
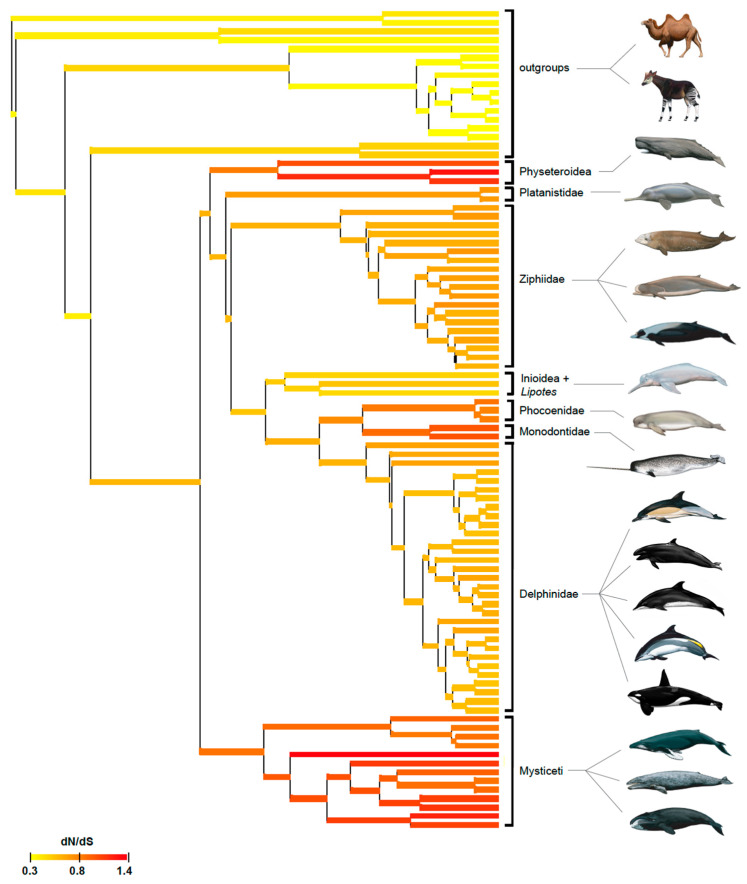
Coevol selection analysis (dN/dS) for 93 taxa. See Figure S8 for a version of this figure with species names for all taxa. Mammal paintings by Carl Buell.

Selection (dN/dS) analyses were performed on a concatenation of seven enamel genes with the Coevol and codeml programs. Figure 4 shows the results of the Coevol selection analysis and provides a visual portrayal of selection intensity on the concatenation of enamel genes in 93 cetartiodactyls. Relaxed purifying selection (red and orange–red branches) is most evident in baleen-bearing mysticetes that lack teeth as adults, but is also apparent in three groups of odontocetes (physeteroids, phocoenids, monodontids) that are characterized by low enamel complexity (Werth scores of 1–3). Further, gradual relaxation of purifying selection on the stem and then crown branches is apparent in both physeteroids and monodontids (Figure 4). Ziphiids that have been investigated also have low Werth scores (1–2), but relaxed purifying selection is less evident in this clade than in the aforementioned odontocetes. By contrast, purifying selection intensity is consistently higher (=lower dN/dS values) in inioids and delphinids that have Werth scores of 4 or 5.

### 3.4. Correlation and Regression Analyses

**Figure 5:**
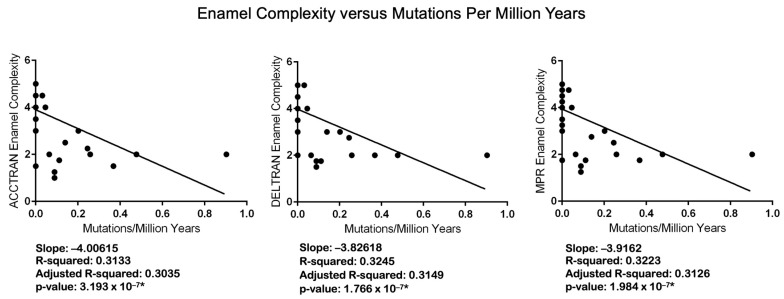
Regression analyses of Werth enamel complexity categories versus counts of mutations per million years for each category. Linear regression analyses were performed with RStudio version 2023.06.2 Build 561 [81] and GraphPad. Asterisks denote statistically significant *p* values.

**Figure 6:**
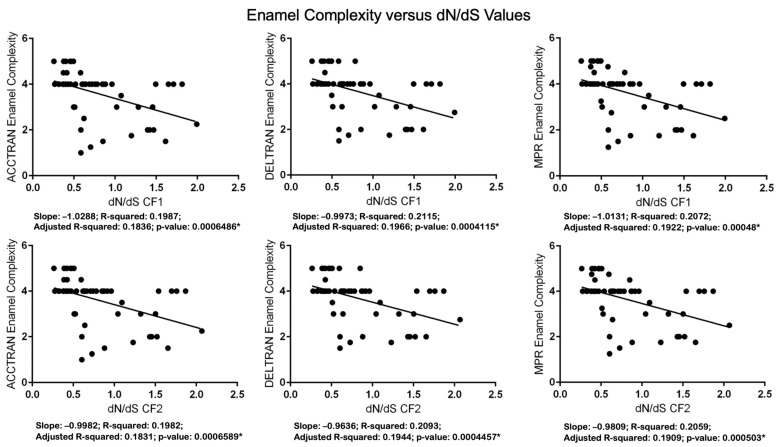
Regression analyses of Werth enamel complexity categories versus dN/dS values with codon frequency models (CF1) and 2 (CF2). Linear regression analyses were performed with RStudio [81] and GraphPad. Asterisks denote statistically significant *p* values.

For 37 taxa, we used Spearman’s rank-order correlation and linear regression to examine the relationships between enamel complexity and two different measures of relaxed selection/neutral evolution in seven enamel genes. The first measure is the number of inactivating mutations/MYRs (Table S4), and the second is dN/dS values that were estimated using codeml (Table S5). The codeml results are based on analyses with a free-ratio model. The results of the correlation analyses are shown in Table 4. The results of the regression analyses are shown in Figure 5 (mutations/MYRs), Figure 6 (dN/dS values), and Figure S6 (log dN/dS values).

All of the results of the correlation analyses are statistically significant (Table 4). *p* values for analyses with mutations/MYRs range from 2.012 × 10^8^ (ACCTRAN) to 4.284 × 10^8^ (DELTRAN). *p* values for analyses with dN/dS values, in turn, range from 2.810 × 10^5^ (ACCTRAN, CF1) to 6.447 × 10^5^ (DELTRAN, CF2). All of the linear regression results are also significant. The regression results for enamel complexity and the number of mutations/MYRs have *p* values that range from 1.766 × 10^7^ (DELTRAN) to 3.193 × 10^7^ (ACCTRAN). *p*-values for six analyses with free-ratio dN/dS values range from *p* = 0.0004115 (DELTRAN, CF1) to *p* = 0.0006589 (ACCTRAN, CF2). We also log-transformed dN/dS values given that Anderson–Darling tests indicated significant departures from normality for the residuals (Table S6). Regression analyses with log-transformed dN/dS values (Figure S6) showed increased significance relative to analyses with non-transformed dN/dS values, and the residuals of these analyses were closer to normality (Table S6). We did not perform analyses with log-transformed mutations/MYRs because most branches have zero mutations and the log of zero is not defined.

## 4. Discussion

### 4.1. Inactivating Mutations in Enamel Genes

Odontocetes display a variety of tooth morphologies and enamel phenotypes. Some river dolphins (i.e., *Platanista* spp., *Inia geoffrensis*) are polydont with highly prismatic enamel, phocoenids are characterized by intermediate enamel with amorphous crystallite aggregations, and narwals (*Monodon monoceros*) usually possess only a single enamelless tooth (“tusk”) that generally erupts only in males in the left upper quadrant of the jaws [4,57]. However, the connection between different enamel phenotypes in odontocetes and the integrity of the underlying genes responsible for proper enamel formation has only been investigated in a limited number of odontocete species. Meredith et al. [31,32] documented inactivating mutations in two enamel genes (*ENAM, MMP20*) in one or both species of *Kogia*, and Mu et al. [33] reported inactivating mutations in *ACP4* in *K. breviceps*. Mu et al. [34], in turn, documented premature stop codons in the *AMELX* and *KLK4* genes of the Yangtze finless porpoise (*Neophocaena asiaeorientalis*). Most recently, Randall et al. [35] examined seven enamel genes in 13 mysticetes and 14 odontocetes and expanded the catalog of inactivating mutations in both groups. In the case of odontocetes, Randall et al. [35] provided evidence for inactivating mutations in four additional genes (*AMBN*, *AMELX, AMTN, KLK4*) in one or both species of *Kogia*. These authors also documented a heterozygous splice site mutation in the *ENAM* gene of killer whale (*Orcinus orca*), a shared splice site mutation in the *AMELX* gene of both monodontids (*Delphinapterus leucas, Monodon monoceros*), autapomorphic inactivating mutations in assorted tooth genes in both monodontids, shared inactivating mutations in the *AMTN* and *KLK4* genes of two phocoenids, and multiple inactivating mutations in the *ACP4* gene of both Sowerby’s beaked whale (*Mesoplodon bidens*) and the sperm whale (*Physeter macrocephalus*).

To further investigate the association between the molecular components of enamel production and the morphology of odontocete enamel, taxon sampling was greatly expanded in the present study to include 63 odontocetes. Parsimony mapping (DELTRAN) documented a total of 48 inactivating mutations in these taxa. The majority of inactivating mutations that were reported in previous studies [31,32,33,34,35] were confirmed here, but there are a few exceptions. First, Mu et al. [34] and Randall et al. [35] both reported a stop codon in exon 6 of *AMELX* in *Neophocaena asiaeorientalis*. However, this stop codon is a CAG (encoding glutamine) instead of a TAG in the individual of *N. asiaeorientalis* that we included in our study. Second, we examined a more complete genome of *Kogia breviceps* than Randall et al. [35] to determine whether we could find any remnants of *ACP4* exons 1–3 and 11 that were reported as missing by Randall et al. [35]. We did not find any remnants of these exons in the second individual of *K. breviceps*. However, a frameshift deletion in exon 7 was present in the individual examined by Randall et al. [35], but not in the individual examined here. Finally, differences in Randall et al.’s [35] 44-taxon alignment for *ACP4* and our 93-taxon alignment for this gene resulted in a slightly upstream location for a 1-bp insertion in exon 10 of *K. breviceps*. This shift also negates a putative stop codon in the ancestral reading frame of this gene.

Among families with Werth enamel complexity scores of 4 or 5 (Delphinidae, Iniidae, Pontoporiidae, Platanistidae), there are only two inactivating mutations in 35 species. One of these mutations is a donor splice site mutation (AT) in intron 4 of the *ENAM* gene in *Orcinus orca* and the second mutation is an in-frame deletion of *AMTN* exon 3 in *Platanista*. In the case of the *ENAM* splice site mutation in *O. orca*, the mutation is present in the assembled genome of this individual (NC_064562), although SRA data (ERS6484570) suggest that this mutation is heterozygous with 18 PacBio reads supporting the AT donor splice site mutation and 17 PacBio reads supporting the canonical GT splice site (Figure S7). We also examined Illumina reads for 13 additional individuals of *O. orca* to determine the frequency of this splice site mutation (Table S7). Among the individuals that we examined, seven were homozygous for the canonical GT splice, three were homozygous for the inactivating mutation (AT), and three are likely heterozygous, exhibiting coverage for both the GT and AT variants. Thus, it appears that this splice site mutation is polymorphic within *Orcinus orca*. Future studies will be required to determine the effect of the AT splice site mutation on the expression of *ENAM* in developing enamel. We note that there is a potential GC splice site that is nine bp downstream from the standard splice site location. This alternate splice site would result in a protein that is three amino acids longer than for the standard splice site. In the case of *Platanista*, we are unaware of alternatively spliced transcripts that lack exon 3, but comparative data are scarce for *AMTN* and it remains possible that *Platanista* species, which have enamel complexity scores of 5, express a variant of *AMTN* that lacks exon 3.

For our analyses, the single species in the family Lipotidae is the recently extinct *Lipotes vexillifer* (Yangtze River dolphin), and this species does not have a Werth enamel complexity score. However, *L. vexillifer* is closely related to the river dolphins *Inia geoffrensis* (Amazon River dolphin) and *Pontoporia blanvillei* (franciscana/La Plata dolphin) that have enamel scores of 5 and 4, respectively. Also, *L. vexillifer* has been reported as having a wrinkled rugose enamel phenotype that is similar to *I. geoffrensis* [13]. The latter species is known to feed on hard-shelled river crabs (*Poppiana*) and river turtles (*Podocnemis*) and to cut large, armored fish and catfish into smaller pieces prior to swallowing [86,87,88]. Perhaps not surprisingly, there are no inactivating mutations in any of the seven enamel genes of *L. vexillifer*. Conversely, it is surprising that the delphinid *Grampus griseus* (Risso’s dolphin) has a Werth enamel complexity score of 4 along with seven intact enamel genes given that this species bears no functional upper teeth and uses suction to feed on soft-bodied cephalopods [4]. One explanation for the presence of prismatic enamel in this species is that there has been insufficient time for inactivating mutations and even replacement substitutions to accumulate. Indeed, Risso’s dolphin is nested phylogenetically among delphinids that retain upper and lower teeth and diverged from its closest extant relatives ~3.9 MYRs ago [69].

By contrast with oceanic and river dolphin families with high Werth enamel complexity scores of 4 or 5, there are 46 inactivating mutations among 27 species that belong to five families with Werth enamel complexity scores of 1, 2, or 3 (Monodontidae, Phocoenidae, Ziphiidae, Kogiidae, Physeteridae). With the exception of Phocoenidae, species belonging to these families are generally characterized by having very few teeth, blunt heads, and feeding strategies that rely exclusively or primarily on suction to obtain soft prey [3,4,89]. Presumably enamel is less important in these taxa that do not require teeth to grasp prey [4]. The complexity of phocoenid enamel, in turn, is intermediate between that of oceanic and river dolphins and that of sperm whales, beaked whales, and monodontids. Phocoenids that have been investigated also employ suction feeding [3] and exhibit intermediate levels of dental occlusion and/or dental contact with prey items [4]. The possible significance of inactivating mutations in enamel genes in these five families is discussed in detail below.

Statistical analyses also support the conclusion that inactivating mutations are more plentiful in lineages with less complex enamel. Specifically, Spearman’s rank-order correlation (Table 4) and linear regression (Figure 5) analyses demonstrate that the number of inactivating mutations/MYRs is negatively correlated with enamel complexity. Odontocete taxa with lower enamel complexity scores have generally accumulated more mutations/MYRs than taxa with higher enamel complexity scores. Selection intensity as measured by dN/dS values is also negatively correlated with enamel complexity based on Spearman’s rank-order correlation (Table 4) and linear regression (Figure 6). Specifically, higher dN/dS values are associated with lower Werth enamel complexity scores and there is a trend of increasing dN/dS values as enamel complexity decreases (Figure 6). The linear regression analyses with dN/dS values are associated with statistically significant non-normality of the residuals, which by itself may be of some concern. However, the results of Spearman’s test, which is nonparametric, corroborate the results of the parametric regression analyses. Also, we log-transformed the dN/dS values and this resulted in stronger statistical significance for the regressions (Figure S6) and weaker violations of the normality tests with the residuals (Table S6). Finally, the normality assumption is less important when N is large (e.g., >30 [90]) because of the central limit theorem [91]. Overall, these results provide compelling evidence for the correlated release of selective constraints at the molecular and phenotypic levels.

### 4.2. Enamel Degeneration in Phocoenidae

Phocoenidae (porpoises) use both ram and suction feeding techniques and have teeth that are mediolaterally compressed and more spade-shaped than dolphins [68]. Phocoenids that have been examined generally have prismless enamel with a low degree of mineralization [5,14], although the narrow-ridged finless porpoise (*Neophocaena phocaenoides*) has been observed to have enamel prisms near the enamel–dentin junction that gradually transition to prismless enamel further away from this junction [5]. Even so, the enamel prisms are simple and interprismatic regions cannot be discerned [5]. The three phocoenids included in our study (*N. asiaeorientalis*, *Phocoena phocoena,* and *Phocoena sinus*) share one inactivating mutation in *AMTN* and a second inactivating mutation in *KLK4*. Interestingly, Núñez et al. [92] examined enamel maturation in *AMTN* and *KLK4* null mice. These authors concluded that AMTN and KLK4 are both essential for proper enamel maturation in mice, and that the absence of both proteins had a more severe effect than the absence of a single protein. Importantly, enamel mineral density is significantly reduced in *AMTN*^−/−^ *KLK4*^−/−^ mice [92]. This finding mirrors the empirical observation that a representative phocoenid (*Phocoena spinipinnis* (Burmeister’s porpoise)) has reduced enamel mineral density relative to oceanic and river dolphins that have intact copies of all seven enamel genes [93].

### 4.3. Enamel Degeneration in Monodontidae

Monodontidae includes two living species, *Monodon monoceros* (narwhal) and *Delphinapterus leucas* (beluga). Most male narwhals exhibit a single enamelless tusk (Werth enamel complexity = 1) that erupts in the upper left quadrant of the jaws, although the smaller right tusk also erupts in some individuals. Female narwhals usually have two unerupted tusks, but, as for males, not all individuals are the same and the left tusk erupts in ~15% of individuals [57]. The beluga has prismless to intermediate enamel (Werth enamel complexity = 2/3). A shared splice site mutation in *AMELX* (Table 3, Figure 3) and the results of selection analyses with Coevol (Figure 4) both suggest that enamel was under relaxed selection in the common ancestor of these two taxa. *AMELX* plays an essential role in enamel formation and prismatic enamel is completely absent in amelogenin-null mice [94]. Stem monodontids may have expressed a modified variant of *AMELX*, but the shared *AMELX* mutation in monodontids is consistent with ancestral reconstructions that suggest enamel had already begun to degenerate in the common ancestor of *Monodon* and *Delphinapterus* (Figures S2–S4). Additional inactivating mutations accumulated independently in two genes in *Delphinapterus* (*ACP4*, *AMTN*) and three genes in *Monodon* (*ACP4*, *AMBN*, *KLK4*) (Table 3, Figure 3). The occurrence of inactivating mutations in a total of three enamel genes in *Delphinapterus* is not surprising given that the enamel in this species has been described as very thin, soft, weakly developed, and largely prismless [4]. Indeed, Werth et al. [4] found that the soft enamel of *Delphinapterus* had the lowest compressive strength of any species whose enamel was tested. In the case of *Monodon*, the inactivation of four enamel genes, including genes that include two enamel matrix proteins (amelogenin, ameloblastin), is expected given that enamel is absent in the narwhal [5]. The enamelless tusk of male narwhals also is used to sense external stimuli in the environment [16]. The evidence for the sensory ability of narwhal tusks includes experimental studies that demonstrate significant changes in heart rate when the external surface of the tusk is exposed to alternating solutions of high-salt and fresh water [16]. Thus, it seems possible that enamel loss may have been advantageous for the development of the sensory functions of the male tusk. Alternate hypotheses for the function of the male narwhal tusk remain speculative but include the detection of (1) waters where females in estrus are gathered, (2) waters where females are foraging, and (3) food sources for calves [17].

### 4.4. Enamel Degeneration in Physeteroidea

Physeteroidea includes Physeteridae and Kogiidae. The former includes *Physeter macrocephalus* (sperm whale) and the latter includes *Kogia breviceps* (pygmy sperm whale) and *K. sima* (dwarf sperm whale). According to Werth et al. (p. 799 in [4]), some individuals of *Physeter* have a “small, thin apical cap of weakly developed prismless enamel.” Cementum is exposed when this enamel cap wears away. This variation among specimens, some that retain a thin enamel cap and others that do not, explains Werth et al.’s [4] enamel score of 1/2 for this species. Other studies have also reported that enamel is either absent [95] or present [5] in *Physeter*. Ishiyama [5] noted that *Physeter* enamel is thicker than the enamel of delphinids that were examined. Ishiyama [5] also found that *Physeter* enamel, like that of *Phocoena phocoena* (harbor porpoise), is pseudoprismatic based on polarizing microscopy and scanning electron microscopy. Unlike prismatic enamel, which possesses both rods (prisms) and interprismatic crystallites, pseudoprismatic enamel includes rods but not interprismatic enamel and is the type of enamel that characterizes most reptiles [8]. For *Kogia breviceps*, Werth et al. [4] examined two individuals, both of which possessed prismless enamel. By contrast, other studies have reported that enamel is absent in *Kogia* [61,96]. This discrepancy may be explained by the presence of a very thin layer of prismless enamel in young individuals of *Kogia* that wears away in life [62,63]. Bianucci and Landini [2] inferred that enamel was absent in the most recent common ancestor of crown Physeteroidea based on their scoring of enamel as absent in both extant genera and several extinct crown physeteroids (*Physeterula, Placoziphius, Orycterocetus*). However, these results are inconsistent with the aforementioned studies that have reported the occurrence of enamel in some specimens of both *Physeter* and *Kogia*. At the molecular level, there are no shared inactivating mutations in *Physeter* and *Kogia,* which is consistent with independent degradation of enamel following the split of these lineages.

*Physeter* only exhibits inactivating mutations in one of seven enamel genes (*ACP4*). Missense mutations in *ACP4* are known to cause hypoplastic enamel in humans [10,97]. There is also a 1-bp frameshift insertion in *ACP4* that causes hypoplastic amelogenesis imperfecta in Akita and American Akita dog breeds [98]. These observations of hypoplastic enamel are consistent with the occurrence of enamel that has apparently worn away in some individuals of *Physeter*. By contrast, the two extant species of Kogiidae have Werth enamel complexity scores of 2 and numerous inactivating mutations. Specifically, there are 19 independent mutations in Kogiidae that are spread across six of the seven enamel genes that we examined. The only unaffected gene is *MMP20*, although Meredith et al. [32] reported a polymorphic premature stop codon in exon 2 of this gene that was present in one of the three *K. breviceps* individuals that they screened. Thus, all seven enamel-specific genes that have been examined possess inactivating mutations within Kogiidae. The wholesale degeneration of enamel genes in *Kogia* species, including four genes with inactivating mutations that are shared by both species, is consistent with the occurrence of very thin enamel that typically wears away by the time that *Kogia* individuals are one year of age [62].

### 4.5. Enamel Degeneration in Ziphiidae

Most extant ziphiid species possess a single pair of teeth in the lower jaw that erupt to become functional only in males. Exceptions include *Berardius* spp. (four-toothed whales), *Hyperoodon* spp. (bottlenose whales), and *Tasmacetus shepherdi* (Shepherd’s beaked whale) [99]. In species of *Berardius*, there are two pairs of teeth that erupt in both sexes. In species of *Hyperoodon*, the single pair of teeth remain unerupted in the gums of both females and males. Finally, *T. shepherdi* differs from other extant ziphiids in having a full set of teeth. Werth enamel complexity scores are only available for three ziphiid species, but in all three cases the scores are low for the erupted teeth, i.e., *Ziphius cavirostris* = 1, *Mesoplodon densirostris* = 1/2, *Berardius bairdii* = 2 [4]. In the case of *Ziphius*, Loch and van Vuuren [100] examined the vestigial teeth that are found in the gums and reported that these teeth are covered by a thin layer of prismless enamel, albeit with pseudoprisms.

Unlike Phocoenidae, Monodontidae, and Physeteroidea, wherein all investigated species have inactivating mutations in one or more enamel genes, only 12 of 19 ziphiids exhibit an inactivating mutation in at least one enamel gene. Six enamel genes exhibit one or more inactivating mutations in this clade, while *KLK4* is intact in all ziphiids. The only shared mutation among ziphiids is a frameshift deletion in the *AMTN* gene that is found in both species of *Hyperoodon*. These are the only beaked whale species in which the teeth remain unerupted in both males and females [99]. Each species of *Hyperoodon* also displays an autapomorphic inactivating mutation in a second enamel gene (*ENAM* in *H. ampullatus*, *AMELX* in *H. planifrons*). Ishiyama [5] described the enamel in *Berardius bairdii* as practically negligible and consisting of fine crystal groups that are arranged perpendicular to the dentinal surface. *B. bairdii* possesses a premature stop codon in exon 5 of the *MMP20* gene, which encodes the enamelysin protein that plays a critical role in the degradation of enamel matrix proteins during the secretory phase of enamel formation. The very thin enamel of *B. bairdii* is consistent with Caterina et al.’s [101] finding that the enamel of *MMP20*-deficient mice is hypoplastic. The second species of *Berardius* (*B. arnuxii*) that we examined also has rudimentary enamel [102] and has inactivating mutations in two EMP genes, *AMELX* and *ENAM*, that encode amelogenin and enamelin, respectively. Amelogenin-null mice have hypoplastic enamel that lacks the prismatic pattern of normal enamel [94]. In the case of enamelin, Hu et al. [36] showed that there is no true enamel in *ENAM* knockin mice. The occurrence of inactivating mutations in both *AMELX* and *ENAM* in *B. arnuxii* is consistent with the rudimentary enamel that is found in this species. The only species of *Mesoplodon* with an enamel complexity score is *M. densirostris* (Werth enamel complexity = 1/2). This species exhibits inactivating mutations in two enamel genes, *ACP4* and *AMTN*. Mutations in *AMTN* are associated with hypomineralized enamel in human clinical cases [44] and a pronounced delay in enamel maturation in *AMTN*-deficient mice [103]. Mutations in *ACP4*, in turn, are associated with hypoplastic enamel in dogs and humans [97,98].

### 4.6. Conclusions and Future Directions

The inactivation of enamel genes has been thoroughly documented in both edentulous taxa (pangolins, anteaters, baleen whales, Steller’s sea cow, birds, turtles) and taxa with enamelless teeth (aardvark, sloths, armadillos) [31,32,35,37,42,43,104]. By contrast with these taxa, extant odontocete species exhibit a broad spectrum of enamel complexities ranging from highly prismatic enamel with HSBs in some river dolphins to the enamelless condition of the single tusk in male narwhals. In between these endpoints are taxa with prismatic enamel but no HSBs (e.g., oceanic dolphins), intermediate enamel (e.g., porpoises), and prismless enamel (e.g., pygmy and dwarf sperm whales). Our examination of seven enamel genes demonstrates that enamel degeneration along this spectrum is underpinned by molecular cavities in the genes that encode enamel development, and that more genes generally are inactivated in taxa with less complex enamel. There are only two inactivating mutations in odontocete taxa with prismatic enamel or prismatic enamel with HSBs, whereas inactivating mutations are much more numerous in taxa with intermediate enamel, prismless enamel, or no enamel at all. Our analysis focused on specific categories of inactivating mutations such as premature stop codons and frameshift indels that can affect the structural integrity and function of the encoded proteins. However, it remains possible that missense mutations (changes from one amino acid to another) in various enamel genes also contribute to the degenerative enamel phenotypes that are found in different odontocetes. In addition, many odontocetes lack information on enamel structure and it will be important to fill in these gaps in our knowledge in future studies. CRISPR-Cas9 studies with reconstructed enamel gene sequences for various odontocete nodes, such as the most recent common ancestor of extant ziphiids, also have the potential to provide key insights into the evolutionary history of enamel degeneration in various clades of odontocetes. Finally, the incorporation of extinct cetaceans into future studies may be informative for determining when transitions between different enamel phenotypes occurred as well as for documenting the origins of tooth and enamel types that are not seen in extant cetaceans [105].

## Figures and Tables

**Table 1:** Taxon sampling for 62 odontocetes, 13 mysticetes, and 17 terrestrial or semiaquatic cetartiodactyl outgroups.

Odontoceti		
Family	Species	Common Name
Delphinidae	*Cephalorhynchus commersonii*	Commerson’s dolphin
Delphinidae	*Cephalorhynchus eutropia*	Black dolphin/Chilean dolphin
Delphinidae	*Cephalorhynchus heavisidii*	Heaviside’s dolphin
Delphinidae	*Cephalorhynchus hectori*	Hector’s dolphin
Delphinidae	*Delphinus delphis bairdii*	Long-beaked common dolphin
Delphinidae	*Delphinus delphis delphis*	Short-beaked dolphin
Delphinidae	*Feresa attenuata*	Pygmy killer whale
Delphinidae	*Globicephala macrorhynchus*	Short-finned pilot whale
Delphinidae	*Globicephala melas*	Long-finned pilot whale
Delphinidae	*Grampus griseus*	Risso’s dolphin
Delphinidae	*Lagenodelphis hosei*	Fraser’s dolphin
Delphinidae	*Lagenorhynchus albirostris*	White-beaked dolphin
Delphinidae	*Leucopleurus acutus*	Atlantic white-sided dolphin
Delphinidae	*Lissodelphis borealis*	Northern right whale dolphin
Delphinidae	*Lissodelphis peronii*	Southern right whale dolphin
Delphinidae	*Orcaella brevirostris*	Irrawaddy dolphin
Delphinidae	*Orcaella heinsohni*	Australian snubfin dolphin
Delphinidae	*Orcinus orca*	Killer whale
Delphinidae	*Peponocephala electra*	Melon-headed whale
Delphinidae	*Pseudorca crassidens*	False killer whale
Delphinidae	*Sagmatias* *obliquidens*	Pacific white-sided dolphin
Delphinidae	*Sagmatias obscurus*	Dusky dolphin
Delphinidae	*Sousa chinensis*	Indo-Pacific humpback dolphin
Delphinidae	*Stenella attenuata*	Pantropical spotted dolphin
Delphinidae	*Stenella clymene*	Clymene dolphin
Delphinidae	*Stenella coeruleoalba*	Striped dolphin
Delphinidae	*Stenella frontalis*	Atlantic spotted dolphin
Delphinidae	*Stenella longirostris*	Eastern spinner dolphin
Delphinidae	*Steno bredanensis*	Rough-toothed dolphin
Delphinidae	*Tursiops aduncus*	Indo-Pacific bottlenose dolphin
Delphinidae	*Tursiops truncatus*	Common bottlenose dolphin
Iniidae	*Inia geoffrensis*	Amazon River dolphin
Kogiidae	*Kogia breviceps*	Pygmy sperm whale
Kogiidae	*Kogia sima*	Dwarf sperm whale
Lipotidae	*Lipotes vexillifer*	Chinese river dolphin/baiji
Monodontidae	*Delphinapterus leucas*	Beluga
Monodontidae	*Monodon monoceros*	Narwhal
Phocoenidae	*Neophocaena asiaeorientalis*	Yangtze finless porpoise
Phocoenidae	*Phocoena phocoena*	Harbor porpoise
Phocoenidae	*Phocoena sinus*	Vaquita
Physeteridae	*Physeter macrocephalus*	Sperm whale
Platanistidae	*Platanista gangetica*	Ganges River dolphin
Platanistidae	*Platanista minor*	Indus River dolphin
Pontoporiidae	*Pontoporia blainvillei*	Franciscana/La Plata dolphin
Ziphiidae	*Berardius arnuxii*	Arnoux’s beaked whale
Ziphiidae	*Berardius bairdii*	Baird’s beaked whale
Ziphiidae	*Hyperoodon ampullatus*	Northern bottlenose whale
Ziphiidae	*Hyperoodon planifrons*	Southern bottlenose whale
Ziphiidae	*Indopacetus pacificus*	Tropical bottlenose whale
Ziphiidae	*Mesoplodon bidens*	Sowerby’s beaked whale
Ziphiidae	*Mesoplodon bowdoini*	Andrew’s beaked whale
Ziphiidae	*Mesoplodon carlhubbsi*	Hubb’s beaked whale
Ziphiidae	*Mesoplodon densirostris*	Blainville’s beaked whale
Ziphiidae	*Mesoplodon europaeus*	Gervais’ beaked whale
Ziphiidae	*Mesoplodon ginkgodens*	Ginkgo-toothed beaked whale
Ziphiidae	*Mesoplodon grayi*	Gray’s beaked whale
Ziphiidae	*Mesoplodon layardii*	Strap-toothed whale
Ziphiidae	*Mesoplodon mirus*	True’s beaked whale
Ziphiidae	*Mesoplodon perrini*	Perrin’s beaked whale
Ziphiidae	*Mesoplodon peruvianus*	Pygmy beaked whale
Ziphiidae	*Mesoplodon stejnegeri*	Stejneger’s beaked whale
Ziphiidae	*Tasmacetus shepherdi*	Shepherd’s beaked whale
Ziphiidae	*Ziphius cavirostris*	Cuvier’s beaked whale
Mysticeti		
Balaenidae	*Balaena mysticetus*	Bowhead whale
Balaenidae	*Eubalaena australis*	Southern right whale
Balaenidae	*Eubalaena glacialis*	North Atlantic right whale
Balaenidae	*Eubalaena japonica*	North Pacific right whale
Balaenopteridae	*Balaenoptera acutorostrata*	Common minke whale
Balaenopteridae	*Balaenoptera bonaerensis*	Antarctic minke whale
Balaenopteridae	*Balaenoptera borealis*	Sei whale
Balaenopteridae	*Balaenoptera musculus*	Blue whale
Balaenopteridae	*Balaenoptera physalus*	Fin whale
Balaenopteridae	*Balaenoptera ricei*	Rice’s whale
Balaenopteridae	*Megaptera novaeangliae*	Humpback whale
Eschrichtiidae	*Eschrichtus robustus*	Gray whale
Neobalaenidae	*Caperea marginata*	Pygmy right whale
Terrestrial and Semiaquatic Cetartiodactyl Outgroups		
Bovidae	*Bison bison*	American bison
Bovidae	*Bos taurus*	Wild yak
Bovidae	*Bubalus bubalis*	Water buffalo
Bovidae	*Capra hircus*	Domestic goat
Bovidae	*Ovis aries*	Domestic sheep
Camelidae	*Camelus bactrianus*	Bactrian camel
Camelidae	*Vicugna pacos*	Alpaca
Cervidae	*Elaphurus davidianus*	Pere David’s deer
Cervidae	*Odocoileus virginianus*	White-tailed deer
Giraffidae	*Giraffa camelopardalis*	Giraffe
Giraffidae	*Okapia johnstoni*	Okapi
Hippopotamidae	*Choeropsis liberiensis*	Pygmy hippopotamus
Hippopotamidae	*Hippopotamus amphibius*	River hippopotamus
Moschidae	*Moschus moschiferus*	Siberian musk deer
Suidae	*Sus scrofa*	Wild boar
Tayassuidae	*Catagonus wagneri*	Chacoan peccary
Tragulidae	*Tragulus javanicus*	Java mouse-deer

**Table 2:** Odontocete and outgroup taxa with a score for Werth enamel complexity.

Odontoceti Species	Werth Enamel Complexity
*Berardius bairdii*	2
*Cephalorhynchus hectori*	4
*Delphinapterus leucas*	2.5 (2/3)
*Delphinus delphis bairdii*	4
*Delphinus delphis delphis*	4
*Globicephala macrorhynchus*	4
*Globicephala melas*	4
*Grampus griseus*	4
*Inia geoffrensis*	5
*Kogia breviceps*	2
*Kogia sima*	2 *
*Lagenodelphis hosei*	4
*Leucopleurus acutus*	4
*Lagenorhynchus albirostris*	4
*Sagmatias obscurus*	4
*Mesoplodon densirostris*	1.5 (1/2)
*Monodon monoceros*	1 *
*Neophocaena asiaeorientalis*	3 *
*Orcaella brevirostris*	4
*Orcinus orca*	4
*Phocoena phocoena*	3
*Physeter macrocephalus*	1.5 (1/2)
*Platanista gangetica*	5
*Platanista minor*	5 *
*Pontoporia blainvillei*	4
*Pseudorca crassidens*	4
*Stenella attenuata*	4
*Stenella clymene*	4
*Stenella coeruleoalba*	4
*Stenella frontalis*	4
*Steno bredanensis*	4
*Tursiops truncatus*	4
*Ziphius cavirostris*	1
**Outgroups**	
*Bos mutus*	5 *
*Camelus bactrianus*	5 *
*Hippopotamus amphibius*	5 *
*Sus scrofa*	5 *

* Denotes species that were assigned a Werth enamel complexity score in this paper. Categories with a decimal are the mean of two categories in parentheses (see main text). 1 = no enamel; 2 = prismless enamel; 3 = intermediate or irregular enamel; 4 = prismatic enamel; and 5 = prismatic enamel with Hunter-Schreger bands or other decussation.

**Table 3:** Inactivating mutations in enamel genes in Odontoceti.

Odontoceti Taxon	Enamel Gene and Mutation
Kogiidae (*K. breviceps + K. sima*)	*ACP4*: E1-3, 11: NBR/NRM; *AMELX*: E2: 47I, In2Do:GG; *ENAM*: E8: 2403D, 3751-3752D; *KLK4*: WGD (NBR/NRM)
Phocoenidae (*N. asiaeorientalis* + *P. phocoena* + *P. sinus*)	*AMTN*: In2Ac:AT; *KLK4*: E2: 73-75S
*Plastanista* (*P. gangetica + P. minor*)	*AMTN*: E3: NBR
*Hyperoodon (H. ampullatus + H. planifrons)*	*AMTN*: E8: 566D
Monodontidae (*D. leucas* + *M. monoceros*)	*AMELX*: In2Do: AT
*Berardius arnuxii*	*AMELX*: E7: NBR/NRM; *ENAM*: E8: 648I
*Berardius bairdii*	*MMP20*: E5:1095-1097S
*Delphinapterus leucas*	*ACP4*: E7: 674D; *AMTN*: E8: 576D
*Hyperoodon ampullatus*	*ENAM*: In6Do: TT
*Hyperoodon planifrons*	*AMELX*: E7: NBR/NRM
*Kogia breviceps*	*ACP4*: E9: 900D; E10: 1100I; *AMBN*: In7Ac: AT; In9Ac: AT; *AMELX*: E2: SCM; *AMTN*: E8: 377D; *ENAM*: In6Do: CT
*Kogia sima*	*AMBN:* WGD (NBR/NRM); *ENAM*: E1-6: NBR/NRM
*Mesoplodon bidens*	*ACP4*: E2: 145-148I, E5: 537-544D, In8Ac: GG
*Mesoplodon densirostris*	*ACP4*: E5: 537-539S (allelic variation); *AMTN*: E5: 249-252I (allelic variation)
*Mesoplodon grayi*	*ACP4*: E8: 802I (allelic variation); *AMELX*: In2Ac: GG/AG (allelic variation)
*Mesoplodon layardii*	*KLK4*: In2Do: CG/GT (allelic variation)
*Mesoplodon perrini*	*AMTN*: E8-9: NRM (no stop)
*Mesoplodon peruvianus*	*AMELX*: E7: NBR/NRM; *AMTN*: E8: 376I
*Monodon monoceros*	*ACP4*: In6Do: AT; *AMBN*: E11: 1214-1216S; *KLK4*: E4: 503-505S
*Orcinus orca*	*ENAM*: In4Do: AT/GT (allelic variation)
*Physeter macrocephalus*	*ACP4*: E4: 427D; E9: 1015D; E10: 1132D (allelic variation)
*Tasmacetus shepherdi*	*KLK4*: E5: 767-769TCM
*Ziphius cavirostris*	*KLK4*: In3Ac: GG

Numbers correspond to positions in the protein-coding sequence alignments (CDS). Abbreviations: Ac = acceptor splice site; D = frameshift deletion; Do = donor splice site; E = exon; I = frameshift insertion; In = intron; NBR = no blast results and possible deletion of exon(s) or gene; NRM = no reads mapped and possible deletion of exon(s) or gene; S = premature stop codon; SCM = start codon mutation; TCM = termination codon mutation.

**Table 4:** Results of Spearman’s rank-order correlation analyses.

X	Y	Rho	*p* Value *
Mutations/MYRs	ACCTRAN	−0.60348	2.01199 × 10^-8^
Mutations/MYRs	DELTRAN	−0.59215	4.28415 × 10^-8^
Mutations/MYRs	MPR	−0.59544	3.44991 × 10^-8^
dN/dS CF1	ACCTRAN	−0.53289	2.80955 × 10^-5^
dN/dS CF2	ACCTRAN	−0.53113	3.01979 × 10^-5^
dN/dS CF1	DELTRAN	−0.51396	5.98125 × 10^-5^
dN/dS CF2	DELTRAN	−0.51202	6.44745 × 10^-5^
dN/dS CF1	MPR	−0.52024	4.67864 × 10^-5^
dN/dS CF2	MPR	−0.51842	5.02687 × 10^-5^

* Two-tailed tests. Abbreviations: CF1, codon frequency model 1; CF2, codon frequency model 2; DELTRAN, delayed transformation; dN/dS, (number of nonsynonymous substitutions/number of nonsynonymous sites)/(number of synonymous substitutions/number of synonymous sites); MPR, most parsimonious reconstruction sets; Mutations, number of inactivating mutations; MYRs, million years; Rho, Spearman’s rank correlation coefficient.

## Data Availability

Data are contained within the article and the Supplementary Materials.

## Supplementary Materials

The following supporting information can be downloaded at: https://www.mdpi.com/article/10.3390/genes15020228/s1: (1) alignments for protein-coding sequences (CDS) for individual enamel genes; (2) maximum likelihood (RAxML) gene trees with bootstrap support values; (3) the 93-taxon species tree for Coevol analysis and 37-taxon species tree and timetree that were used for ancestral reconstructions, codeml analyses, and calculations of the number of inactivating mutations/MYRs for different enamel phenotypes; (4) files for Coevol analyses; (5) files for codeml analyses; (6) files for regression analyses; (7) accession numbers for enamel gene sequences (Table S1); (8) SRA coverage for autapomorphic inactivating mutations (Table S2); (9) well-supported clades on gene trees (Table S3); (10) number of inactivating mutations/MYRs and Werth enamel complexity scores for branches on the rooted species tree for 37-taxa (Table S4); (11) results of free-ratio dN/dS analyses with codeml and Werth enamel complexity scores for branches on the unrooted species tree for 37-taxa (Table S5); (12) normality test results and residuals from linear regression analyses (Table S6); (13) SRA splice site coverage for the *ENAM* intron splice site mutation in *Orcinus orca* (Table S7); (14) node numbers for the 37-taxon tree (Figure S1); (15) Mesquite ancestral reconstruction of enamel phenotypes (Figure S2); (16) ACCTRAN ancestral reconstruction of enamel phenotypes (Figure S3); (17) DELTRAN ancestral reconstruction of enamel phenotypes (Figure S4); (18) *ACP4* exon deletions in *Kogia breviceps* (Figure S5); (19) linear regression results with log-transformed dN/dS values (Figure S6); (20) heterozygous splice site mutation in *ENAM* in the RefSeq genome for *Orcinus orca* (Figure S7); and (21) the results of Coevol analysis with species names for all taxa (Figure S8). Refs. [106,107,108].
