# Supplementary material for: Molecular Evidence for Relaxed Selection on the Enamel Genes of Toothed Whales (Odontoceti) with Degenerative Enamel Phenotypes
Source: Genes (Basel). 2024 Feb 10;15(2):228. doi: 10.3390/genes15020228 (PMC10888366; doi:10.3390/genes15020228)
Supplement: Supplementary file 1 [file genes-15-00228-s001.zip › Supplementary Materials/Supplementary Figures/Figure S5 (ACP4 exon deletions).pdf]

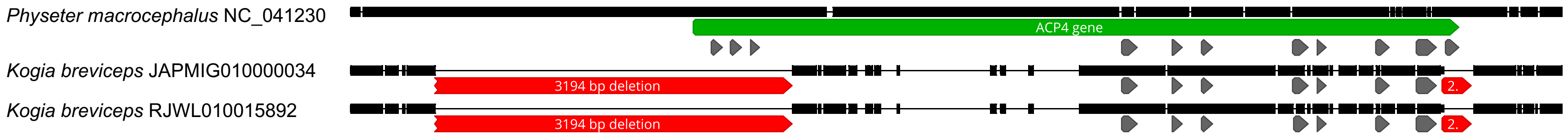

Figure S5. Alignment of partial scaffolds of the RefSeq genome for *Physeter macrocephalus* (sperm whale) and two different individuals of *Kogia breviceps* (pygmy sperm whale) to show exon deletions in the *ACP4* gene of *K. breviceps*. The 5' deletion is 3194 bp and encompasses exons 1-3. The 3' deletion in is 269 bp and encompasses exon 11. The *ACP4* gene in *P. macrocephalus* is denoted in green. Protein-coding exons are shown in gray. There are 11 protein-coding exons in *P. macrocephalus*. Deletions in *K. breviceps* than encompass entire exons are shown in red.
