# Supplementary material for: Molecular Evidence for Relaxed Selection on the Enamel Genes of Toothed Whales (Odontoceti) with Degenerative Enamel Phenotypes
Source: Genes (Basel). 2024 Feb 10;15(2):228. doi: 10.3390/genes15020228 (PMC10888366; doi:10.3390/genes15020228)
Supplement: Supplementary file 1 [file genes-15-00228-s001.zip › Supplementary Materials/Supplementary Figures/Figure S7 (Orcinus splice site mutation).pdf]

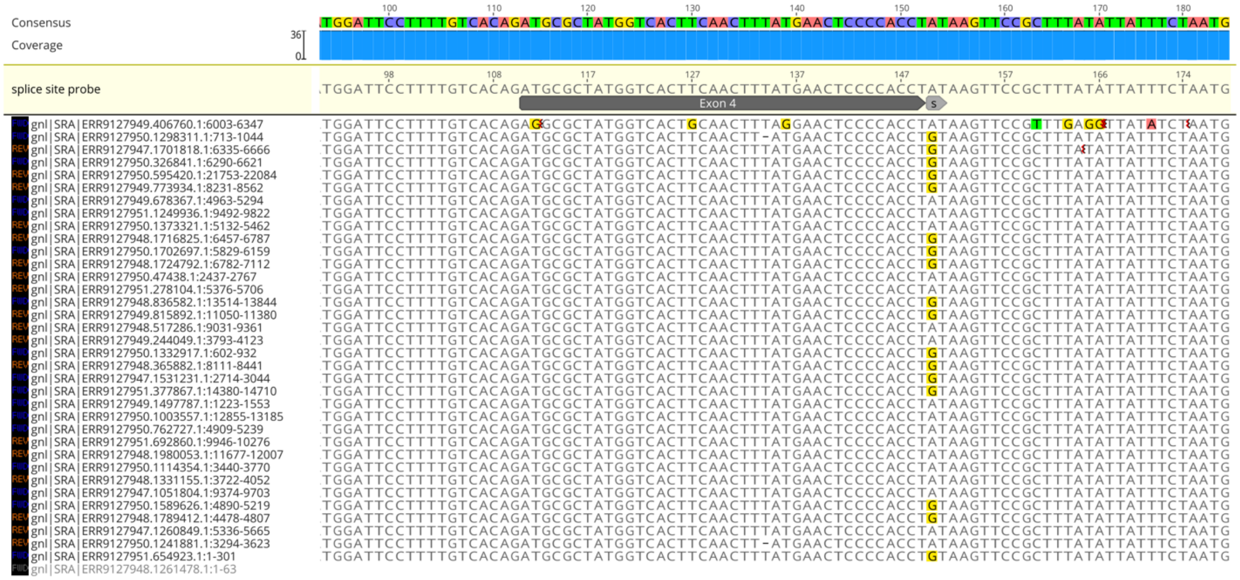

Figure S7. PacBio coverage from ERS6484570 for the intron 4 donor splice site of the *ENAM* gene in *Orcinus orca*. Eighteen reads support the splice site mutation (AT) and 17 reads support the canonical splice site (GT). ERS6484570 corresponds to the RefSeq genome for *O. orca* (GCF_937001465.1). The assembled RefSeq genome based on ERS6484570 contains the donor splice site mutation (see accession NC_064562).
