# Supplementary material for: Molecular Evidence for Relaxed Selection on the Enamel Genes of Toothed Whales (Odontoceti) with Degenerative Enamel Phenotypes
Source: Genes (Basel). 2024 Feb 10;15(2):228. doi: 10.3390/genes15020228 (PMC10888366; doi:10.3390/genes15020228)
Supplement: Supplementary file 1 [file genes-15-00228-s001.zip › Supplementary Materials/Supplementary Figures/Figure S3 (ACCTRAN ancestral reconstruction).pdf]

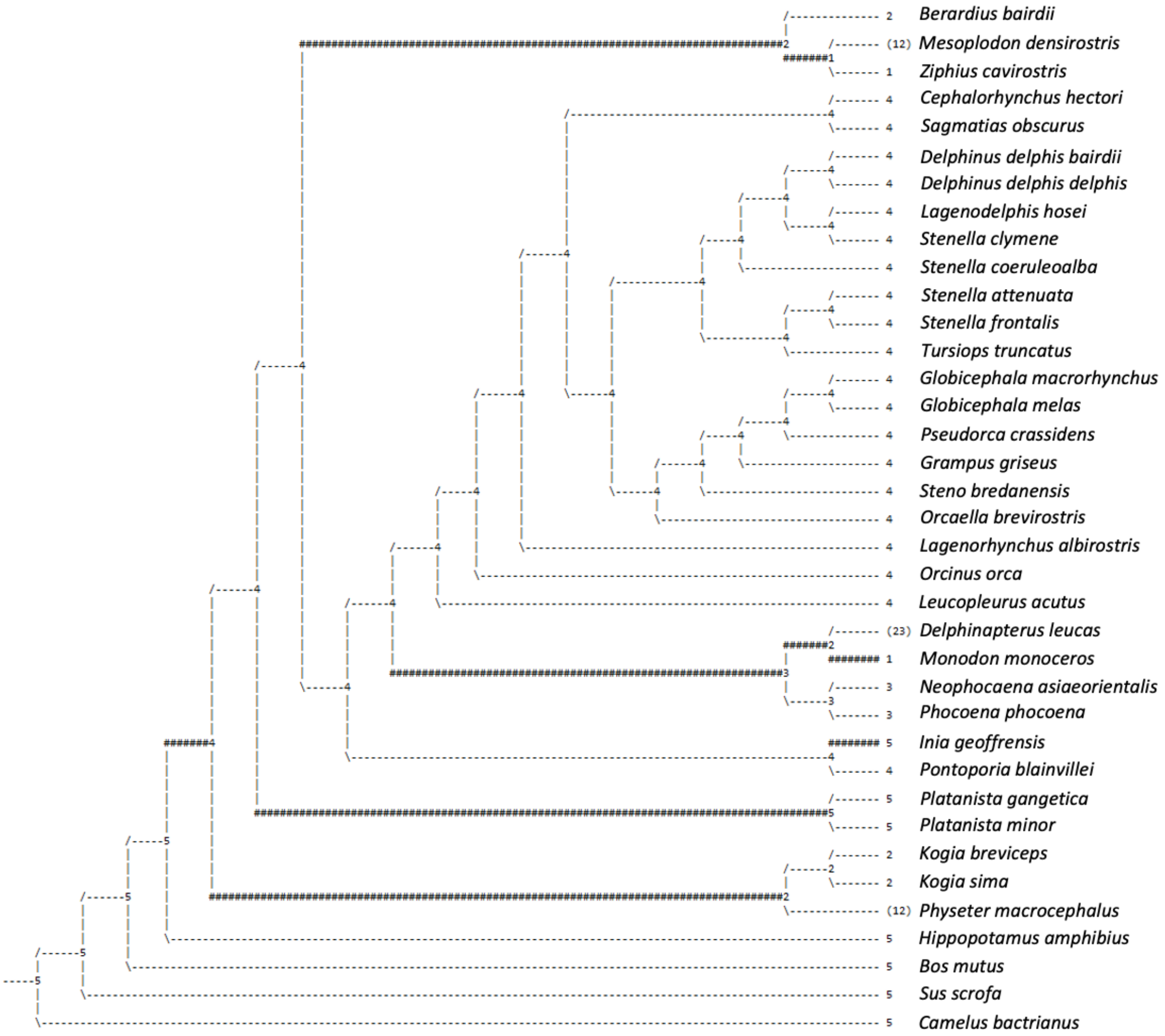

Figure S3. Ancestral reconstruction of Werth Enamel Complexity with the accelerated transformation (ACCTRAN) character optimization option of PAUP*. Branches drawn with pound signs (#) indicate character state transformations. Taxa with two states are polymorphic.
