# Supplementary material for: Molecular Evidence for Relaxed Selection on the Enamel Genes of Toothed Whales (Odontoceti) with Degenerative Enamel Phenotypes
Source: Genes (Basel). 2024 Feb 10;15(2):228. doi: 10.3390/genes15020228 (PMC10888366; doi:10.3390/genes15020228)
Supplement: Supplementary file 1 [file genes-15-00228-s001.zip › Supplementary Materials/Supplementary Figures/Figure S6 (regression with log dN:dS).pdf]

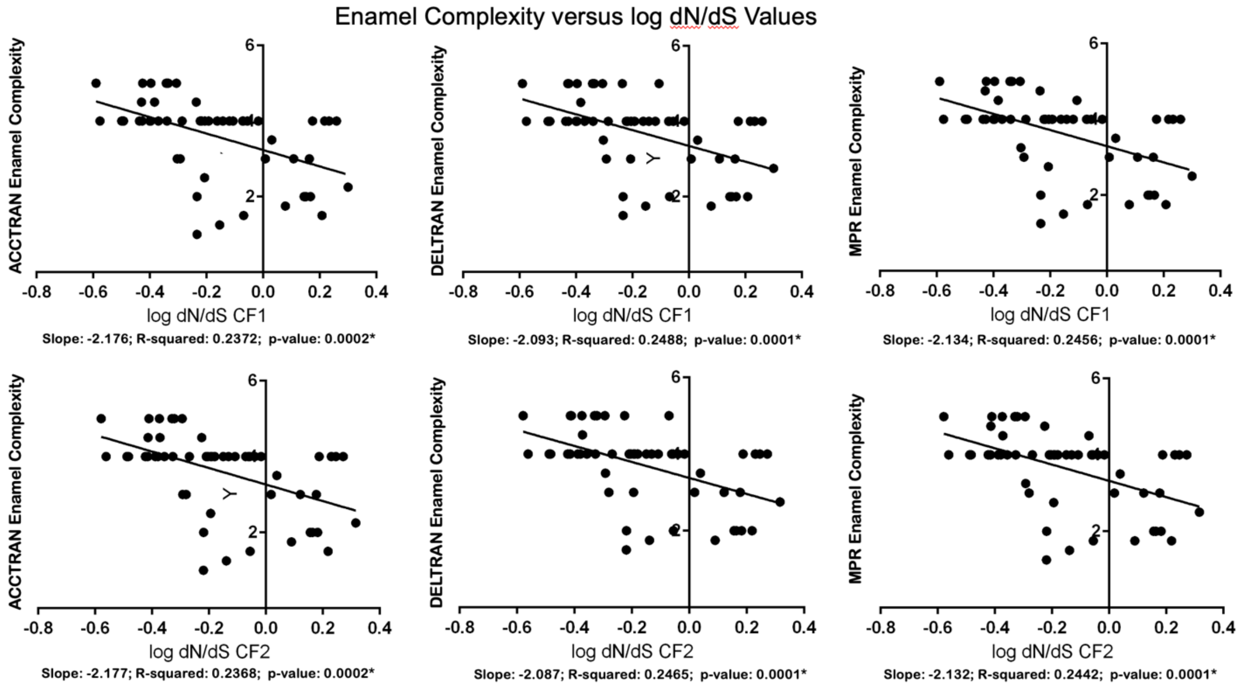

Figure S6. Regression analyses of Werth Enamel Complexity categories versus log-transformed dN/dS values with codon frequency models (CF1) and 2 (CF2). Linear regression analyses were performed with GraphPad. Asterisks denote statistically significant p values.
