# Supplementary material for: Molecular Evidence for Relaxed Selection on the Enamel Genes of Toothed Whales (Odontoceti) with Degenerative Enamel Phenotypes
Source: Genes (Basel). 2024 Feb 10;15(2):228. doi: 10.3390/genes15020228 (PMC10888366; doi:10.3390/genes15020228)
Supplement: Supplementary file 1 [file genes-15-00228-s001.zip › Supplementary Materials/Supplementary Figures/Figure S2 (Mesquite ancestral reconstruction).pdf]

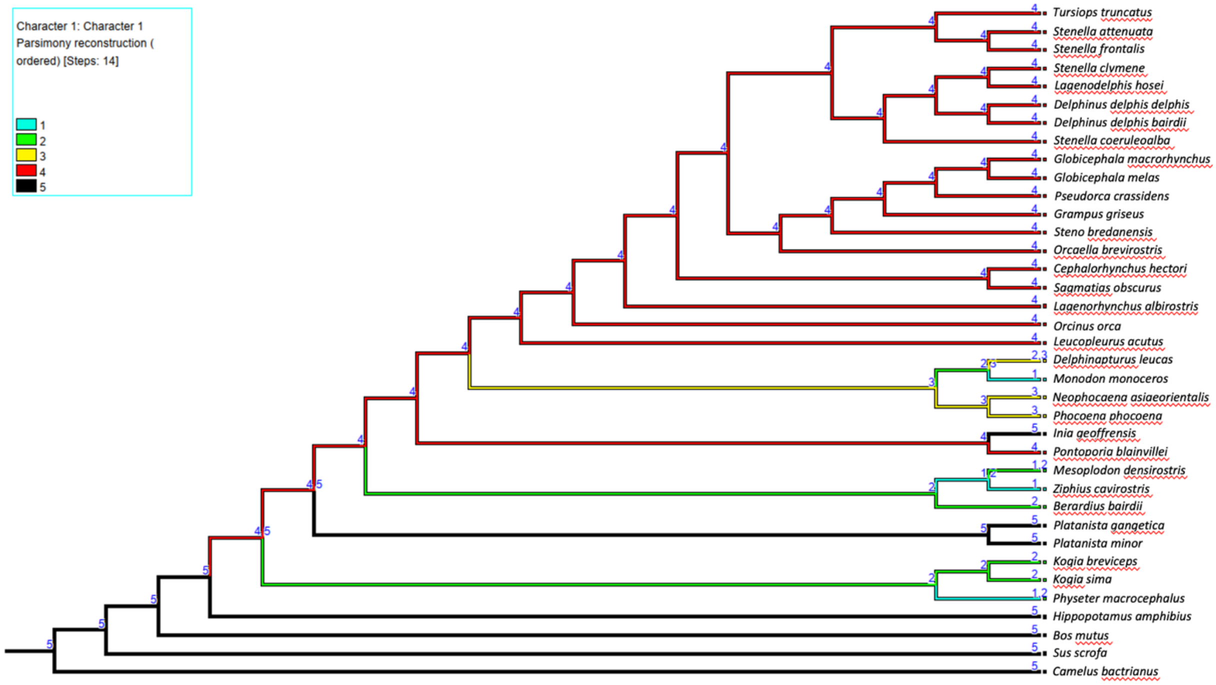

Figure S2. Ancestral reconstruction of Werth Enamel Complexity based on Mesquite and PAUP* with the MPR model. Colors represent Werth Enamel Complexity scores as shown in the legend. Taxa with two states are polymorphic.
