# Supplementary material for: Molecular Evidence for Relaxed Selection on the Enamel Genes of Toothed Whales (Odontoceti) with Degenerative Enamel Phenotypes
Source: Genes (Basel). 2024 Feb 10;15(2):228. doi: 10.3390/genes15020228 (PMC10888366; doi:10.3390/genes15020228)
Supplement: Supplementary file 1 [file genes-15-00228-s001.zip › Supplementary Materials/Supplementary Tables/Table S4 (mutations per million years).docx]

**Table S4.** Mutations per million years and Werth Enamel Complexity scores for branches on the rooted species tree for 37 taxa. Node numbers correspond to Figure S1.

| **Branch (Basal Node Apical Node)** | **Mutations per Million Years** | **Werth Enamel Complexity Reconstruction** | | |
| --- | --- | --- | --- | --- |
|  |  | **ACCTRAN** | **DELTRAN** | **MPR** |
| 58 🡪 *Berardius bairdii* | 0.0641 | 2 | 2 | 2 |
| 42 🡪 *Cephalorhynchus hectori* | 0 | 4 | 4 | 4 |
| 49 🡪 *Delphinapterus leucas* | 0.2454 | 2.25 | 2.75 | 2.5 |
| 27 🡪 *Delphinus delphis bairdii* | 0 | 4 | 4 | 4 |
| 27 🡪 *Delphinus delphis delphis* | 0 | 4 | 4 | 4 |
| 35 🡪 *Globicephala macrorhynchus* | 0 | 4 | 4 | 4 |
| 35 🡪 *Globicephala melas* | 0 | 4 | 4 | 4 |
| 33 🡪 *Grampus griseus* | 0 | 4 | 4 | 4 |
| 55 🡪 *Inia geoffrensis* | 0 | 4.5 | 4.5 | 4.5 |
| 67 🡪 *Kogia breviceps* | 0.9032 | 2 | 2 | 2 |
| 67 🡪 *Kogia sima* | 0.2581 | 2 | 2 | 2 |
| 24 🡪 *Lagenodelphis hosei* | 0 | 4 | 4 | 4 |
| 11 🡪 *Leucopleurus acutus* | 0 | 4 | 4 | 4 |
| 13 🡪 *Lagenorhynchus albirostris* | 0 | 4 | 4 | 4 |
| 42 🡪 *Sagmatias obscurus* | 0 | 4 | 4 | 4 |
| 59 🡪 *Mesoplodon densirostris* | 0.0893 | 1.25 | 1.75 | 1.5 |
| 49 🡪 *Monodon monoceros* | 0.3681 | 1.5 | 2 | 1.75 |
| 52 🡪 *Neophocaena asiaeorientalis* | 0 | 3 | 3 | 3 |
| 31 🡪 *Orcaella brevirostris* | 0 | 4 | 4 | 4 |
| 12 🡪 *Orcinus orca* | 0.0457 | 4 | 4 | 4 |
| 52 🡪 *Phocoena phocoena* | 0 | 3 | 3 | 3 |
| 66 🡪 *Physeter macrocephalus* | 0.1115 | 1.75 | 1.75 | 1.75 |
| 63 🡪 *Platanista gangetica* | 0 | 5 | 5 | 5 |
| 63 🡪 *Platanista minor* | 0 | 5 | 5 | 5 |
| 55 🡪 *Pontoporia blainvillei* | 0 | 4 | 4 | 4 |
| 34 🡪 *Pseudorca crassidens* | 0 | 4 | 4 | 4 |
| 19 🡪 *Stenella attenuata* | 0 | 4 | 4 | 4 |
| 24 🡪 *Stenella clymene* | 0 | 4 | 4 | 4 |
| 22 🡪 *Stenella coeruleoalba* | 0 | 4 | 4 | 4 |
| 19 🡪 *Stenella frontalis* | 0 | 4 | 4 | 4 |
| 32 🡪 *Steno bredanensis* | 0 | 4 | 4 | 4 |
| 17 🡪 *Tursiops truncatus* | 0 | 4 | 4 | 4 |
| 59 🡪 *Ziphius cavirostris* | 0.0893 | 1 | 1.5 | 1.25 |
| 4 🡪 *Bos mutus* | 0 | 5 | 5 | 5 |
| 2 🡪 *Camelus bactrianus* | 0 | 5 | 5 | 5 |
| 5 🡪 *Hippopotamus amphibius* | 0 | 5 | 5 | 5 |
| 3 🡪 *Sus scrofa* | 0 | 5 | 5 | 5 |
| 2 🡪 3 | 0 | 5 | 5 | 5 |
| 3 🡪 4 | 0 | 5 | 5 | 5 |
| 4 🡪 5 | 0 | 5 | 5 | 5 |
| 5 🡪 6 | 0 | 4.5 | 5 | 4.75 |
| 6 🡪 66 | 0 | 3 | 3.5 | 3.25 |
| 66 🡪 67 | 0.4772 | 2 | 2 | 2 |
| 6 🡪 7 | 0 | 4 | 5 | 4.5 |
| 7 🡪 63 | 0.0313 | 4.5 | 5 | 4.75 |
| 7 🡪 8 | 0 | 4 | 4.5 | 4.25 |
| 8 🡪 58 | 0 | 3 | 3 | 3 |
| 58 🡪 59 | 0 | 1.5 | 2 | 1.75 |
| 8 🡪 9 | 0 | 4 | 4 | 4 |
| 9 🡪 55 | 0 | 4 | 4 | 4 |
| 9 🡪 10 | 0 | 4 | 4 | 4 |
| 10 🡪 48 | 0 | 3.5 | 3.5 | 3.5 |
| 48 🡪 49 | 0.1395 | 2.5 | 3 | 2.75 |
| 48 🡪 52 | 0.2020 | 3 | 3 | 3 |
| 10 🡪 11 | 0 | 4 | 4 | 4 |
| 11 🡪 12 | 0 | 4 | 4 | 4 |
| 12 🡪 13 | 0 | 4 | 4 | 4 |
| 13 🡪 14 | 0 | 4 | 4 | 4 |
| 14 🡪 42 | 0 | 4 | 4 | 4 |
| 14 🡪 15 | 0 | 4 | 4 | 4 |
| 15 🡪 31 | 0 | 4 | 4 | 4 |
| 31 🡪 32 | 0 | 4 | 4 | 4 |
| 32 🡪 33 | 0 | 4 | 4 | 4 |
| 33 🡪 34 | 0 | 4 | 4 | 4 |
| 34 🡪 35 | 0 | 4 | 4 | 4 |
| 15 🡪 16 | 0 | 4 | 4 | 4 |
| 16 🡪 22 | 0 | 4 | 4 | 4 |
| 22 🡪 23 | 0 | 4 | 4 | 4 |
| 23 🡪 24 | 0 | 4 | 4 | 4 |
| 23 🡪 27 | 0 | 4 | 4 | 4 |
| 16 🡪 17 | 0 | 4 | 4 | 4 |
| 17 🡪 19 | 0 | 4 | 4 | 4 |

Abbreviations: ACCTRAN, accelerated transformation; DELTRAN, delayed transformation optimization; MPR, most parsimonious reconstruction sets in PAUP (equivalent to reconstructions in Mesquite).
