# Supplementary material for: Molecular Evidence for Relaxed Selection on the Enamel Genes of Toothed Whales (Odontoceti) with Degenerative Enamel Phenotypes
Source: Genes (Basel). 2024 Feb 10;15(2):228. doi: 10.3390/genes15020228 (PMC10888366; doi:10.3390/genes15020228)
Supplement: Supplementary file 1 [file genes-15-00228-s001.zip › Supplementary Materials/Supplementary Tables/Table S3 (well supported clades).docx]

**Table S3.** Monophyly of well-supported cetartiodactyl clades on maximum likelihood phylograms for seven enamel related genes.

| Clade |  |  |  | Gene |  |  |  |
| --- | --- | --- | --- | --- | --- | --- | --- |
|  | ***ACP4*** | ***AMBN*** | ***AMELX*** | ***AMTN*** | ***ENAM*** | ***KLK4*** | ***MMP20*** |
| Delphinidae | yes | no | yes | yes | yes | yes | polytomy |
| Phocoenidae | yes | yes | yes | yes | yes | yes | yes |
| Monodontidae | yes | yes | no | yes | yes | yes | yes |
| Phocoenidae + Monodontidae | yes | yes | no | yes | yes | no | no |
| Delphinoidea | yes | yes | yes | yes | yes | yes | yes |
| Delphinida | yes | yes | yes | yes | yes | yes | polytomy |
| Inioidea | yes | yes | yes | yes | yes | yes | yes |
| Iniidae + Pontoporiidae + Lipotidae | no | no | yes | no | yes | no | yes |
| Ziphiidae | yes | yes | yes | yes | yes | yes | yes |
| Delphinida + Ziphiidae | yes | yes | no | polytomy | yes | yes | yes |
| Platanistidae | yes | yes | yes | yes | yes | yes | yes |
| Kogiidae | yes | **n/a** | yes | yes | yes | **n/a** | yes |
| Physeteroidea | no | yes | yes | yes | yes | **n/a** | yes |
| Odontoceti | no | yes | no | no | yes | yes | yes |
| Balaenopteroidea | no | no | yes | no | yes | yes | yes |
| Balaenidae | yes | yes | yes | yes | yes | yes | yes |
| Mysticeti | yes | yes | yes | no | yes | yes | yes |
| Cetacea | yes | yes | yes | yes | yes | yes | yes |
| Hippopotamidae | yes | yes | yes | yes | yes | yes | yes |
| Cetancodonta* | yes | no | no | yes | yes | no | yes |
| Cetancodonta + Ruminantia | yes | yes | no | yes | yes | yes | yes |
| Giraffidae | yes | yes | no | yes | yes | yes | yes |
| Cervidae | yes | yes | yes | yes | yes | yes | yes |
| Caprinae | yes | yes | yes | yes | yes | yes | yes |
| Bovinae | yes | yes | yes | no | yes | yes | yes |
| Bovidae | yes | no | no | yes | yes | yes | yes |
| Pecora | yes | yes | yes | yes | yes | yes | yes |
| Ruminantia | yes | yes | yes | yes | yes | yes | yes |
| Suina** | yes | yes | yes | yes | yes | yes | yes |
| Camelidae | yes | yes | yes | yes | yes | yes | yes |
| Total number of clades recovered | 26/30 | 24/29 | 22/30 | 24/30 | 30/30 | 25/28 | 27/30 |
| Percentage of clades recovered | 86.67% | 82.8% | 73.3% | 80% | 100% | 89.29% | 90% |

*Same as Whippomorpha (Waddell et al., 1999), **sensu Spaulding et al. (1999) and Hassanin et al. (2012).

Delphinoidea = Delphinidae + Phocoenidae + Monodontidae; Delphinida = Inioidea + Lipotidae + Delphinoidea; Inioidea = Iniidae + Pontoporiidae; Physeteroidea = Kogiidae + Physeteridae.

**References**

Hassanin, A.; Delsuc, F.; Ropiquet, A.; Hammer, C.; Jansen Van Vuuren, B.; Matthee, C.; Ruiz-Garcia, M.; Catzeflis, F.; Areskoug, V.; Nguyen, T.T.; et al. Pattern and timing of diversification of Cetartiodactyla (Mammalia, Laurasiatheria), as revealed by a comprehensive analysis of mitochondrial genomes. *Comptes Rendus - Biol.* **2012**, *335*, 32–50.

Spaulding, M.; O’Leary, M.A.; Gatesy, J. Relationships of Cetacea (Artiodactyla) among mammals: increased taxon sampling alters interpretations of key fossils and character evolution. *PLoS ONE* **2009**, *4*, e7062.

Waddell, P.J.; Okada, N.; Hasegawa, M. Towards resolving the interordinal relationships of placental mammals. *Syst. Biol.* **1999**, *48*, 1–5.
