# Supplementary material for: Molecular Evidence for Relaxed Selection on the Enamel Genes of Toothed Whales (Odontoceti) with Degenerative Enamel Phenotypes
Source: Genes (Basel). 2024 Feb 10;15(2):228. doi: 10.3390/genes15020228 (PMC10888366; doi:10.3390/genes15020228)
Supplement: Supplementary file 1 [file genes-15-00228-s001.zip › Supplementary Materials/Supplementary Tables/Table S5 (free ratio dN:dS).docx]

**Table S5.** Results of selection analyses (dN/dS) with the free-ratio model and ancestral reconstructions of Werth Enamel Complexity with three character optimization methhods (ACCTRAN, DELTRAN, MPR) for branches on the unrooted species tree for 37 taxa. Selection results were obtained with the codeml program of PAML (Yang, 2007). Node numbers correspond to Figure S1.

| **Branch (Basal Node Apical Node)** | **dN/dS Analysis** | | **Werth Enamel Complexity Reconstruction** | | |
| --- | --- | --- | --- | --- | --- |
|  | **CF1** | **CF2** | **ACCTRAN** | **DELTRAN** | **MPR** |
| 58 🡪 *Berardius bairdii* | 0.5838 | 0.6037 | 2 | 2 | 2 |
| 42 🡪 *Cephalorhynchus hectori* | 1.8143 | 1.8691 | 4 | 4 | 4 |
| 49 🡪 *Delphinapterus leucas* | 1.9924 | 2.0679 | 2.25 | 2.75 | 2.5 |
| 27 🡪 *Delphinus delphis bairdii* | 1.2815 | 1.3197 | 4 | 4 | 4 |
| 27 🡪 *Delphinus delphis delphis* | 0.0001 | 0.0001 | 4 | 4 | 4 |
| 35 🡪 *Globicephala macrorhynchus* | 1.7108 | 1.7602 | 4 | 4 | 4 |
| 35 🡪 *Globicephala melas* | 1.6479 | 1.6977 | 4 | 4 | 4 |
| 33 🡪 *Grampus griseus* | 0.427 | 0.4399 | 4 | 4 | 4 |
| 55 🡪 *Inia geoffrensis* | 0.4136 | 0.4245 | 4.5 | 4.5 | 4.5 |
| 67 🡪 *Kogia breviceps* | 1.3951 | 1.4311 | 2 | 2 | 2 |
| 67 🡪 *Kogia sima* | 1.4183 | 1.4545 | 2 | 2 | 2 |
| 24 🡪 *Lagenodelphis hosei* | 0.8513 | 0.8776 | 4 | 4 | 4 |
| 11 🡪 *Leucopleurus acutus* | 0.7601 | 0.7796 | 4 | 4 | 4 |
| 13 🡪 *Lagenorhynchus albirostris* | 0.8471 | 0.8673 | 4 | 4 | 4 |
| 42 🡪 *Sagmatias obscurus* | 0.6422 | 0.6616 | 4 | 4 | 4 |
| 59 🡪 *Mesoplodon densirostris* | 0.7023 | 0.7266 | 1.25 | 1.75 | 1.5 |
| 49 🡪 *Monodon monoceros* | 1.6131 | 1.6532 | 1.5 | 2 | 1.75 |
| 52 🡪 *Neophocaena asiaeorientalis* | 0.5094 | 0.5248 | 3 | 3 | 3 |
| 31 🡪 *Orcaella brevirostris* | 0.4011 | 0.409 | 4 | 4 | 4 |
| 12 🡪 *Orcinus orca* | 0.6226 | 0.6456 | 4 | 4 | 4 |
| 52 🡪 *Phocoena phocoena* | 1.2821 | 1.3209 | 3 | 3 | 3 |
| 66 🡪 *Physeter macrocephalus* | 1.1976 | 1.2287 | 1.75 | 1.75 | 1.75 |
| 63 🡪 *Platanista gangetica* | 999 | 999 | 5 | 5 | 5 |
| 63 🡪 *Platanista minor* | 999 | 999 | 5 | 5 | 5 |
| 55 🡪 *Pontoporia blainvillei* | 0.5175 | 0.5385 | 4 | 4 | 4 |
| 34 🡪 *Pseudorca crassidens* | 1.4942 | 1.5393 | 4 | 4 | 4 |
| 19 🡪 *Stenella attenuata* | 1.2889 | 1.3251 | 4 | 4 | 4 |
| 24 🡪 *Stenella clymene* | 0.3656 | 0.3768 | 4 | 4 | 4 |
| 22 🡪 *Stenella coeruleoalba* | 999 | 999 | 4 | 4 | 4 |
| 19 🡪 *Stenella frontalis* | 0.3735 | 0.3848 | 4 | 4 | 4 |
| 32 🡪 *Steno bredanensis* | 0.8809 | 0.9148 | 4 | 4 | 4 |
| 17 🡪 *Tursiops truncatus* | 0.6064 | 0.6262 | 4 | 4 | 4 |
| 59 🡪 *Ziphius cavirostris* | 0.5841 | 0.6028 | 1 | 1.5 | 1.25 |
| 4 🡪 *Bos mutus* | 0.4543 | 0.4676 | 5 | 5 | 5 |
| 3 🡪 *Camelus bactrianus* | 0.3755 | 0.3881 | 5 | 5 | 5 |
| 5 🡪 *Hippopotamus amphibius* | 0.4941 | 0.5076 | 5 | 5 | 5 |
| 3 🡪 *Sus scrofa* | 0.4623 | 0.4768 | 5 | 5 | 5 |
| 3 🡪 4 | 0.2568 | 0.2638 | 5 | 5 | 5 |
| 4 🡪 5 | 0.4014 | 0.4222 | 5 | 5 | 5 |
| 5 🡪 6 | 0.3716 | 0.3853 | 4.5 | 5 | 4.75 |
| 6 🡪 66 | 0.4969 | 0.5105 | 3 | 3.5 | 3.25 |
| 66 🡪 67 | 1.4684 | 1.5198 | 2 | 2 | 2 |
| 6 🡪 7 | 0.7831 | 0.8492 | 4 | 5 | 4.5 |
| 7 🡪 63 | 0.5799 | 0.5949 | 4.5 | 5 | 4.75 |
| 7 🡪 8 | 0.6541 | 0.6936 | 4 | 4.5 | 4.25 |
| 8 🡪 58 | 1.0182 | 1.0434 | 3 | 3 | 3 |
| 58 🡪 59 | 0.8527 | 0.8794 | 1.5 | 2 | 1.75 |
| 8 🡪 9 | 0.3975 | 0.4175 | 4 | 4 | 4 |
| 9 🡪 55 | 0.3163 | 0.3247 | 4 | 4 | 4 |
| 9 🡪 10 | 0.6243 | 0.6397 | 4 | 4 | 4 |
| 10 🡪 48 | 1.073 | 1.092 | 3.5 | 3.5 | 3.5 |
| 48 🡪 49 | 0.6213 | 0.6396 | 2.5 | 3 | 2.75 |
| 48 🡪 52 | 1.4556 | 1.5021 | 3 | 3 | 3 |
| 10 🡪 11 | 0.6881 | 0.709 | 4 | 4 | 4 |
| 11 🡪 12 | 0.3215 | 0.3288 | 4 | 4 | 4 |
| 12 🡪 13 | 301.7 | 0.4807 | 4 | 4 | 4 |
| 13 🡪 14 | 0.1815 | 0.1878 | 4 | 4 | 4 |
| 14 🡪 42 | 0.2653 | 0.2745 | 4 | 4 | 4 |
| 14 🡪 15 | 0.6412 | 0.6607 | 4 | 4 | 4 |
| 15 🡪 31 | 999 | 999 | 4 | 4 | 4 |
| 31 🡪 32 | 999 | 999 | 4 | 4 | 4 |
| 32 🡪 33 | 0.9622 | 0.9623 | 4 | 4 | 4 |
| 33 🡪 34 | 999 | 1.3263 | 4 | 4 | 4 |
| 34 🡪 35 | 0.4574 | 0.4713 | 4 | 4 | 4 |
| 15 🡪 16 | 0.0001 | 0.0001 | 4 | 4 | 4 |
| 16 🡪 22 | 999 | 999 | 4 | 4 | 4 |
| 22 🡪 23 | 0.5997 | 0.6172 | 4 | 4 | 4 |
| 23 🡪 24 | 0.0001 | 0.0001 | 4 | 4 | 4 |
| 23 🡪 27 | 0.6406 | 0.6601 | 4 | 4 | 4 |
| 16 🡪 17 | 999 | 1.1069 | 4 | 4 | 4 |
| 17 🡪 19 | 0.7202 | 0.7394 | 4 | 4 | 4 |

Abbreviations: ACCTRAN, accelerated transformation; All, all reconstruction methods; CF1, codon frequency model 1; CF2, codon frequency model 2; DELTRAN, delayed transformation optimization; MPR, most parsimonious reconstruction sets in PAUP (equivalent to reconstructions in Mesquite). Codeml dN/dS values for 16 branches are based on less than five nonsynonymous + synonymous substitutions (in some cases zero nonsynonymous + synonymous substitutions) and are shown in purple font. These small sample size estimates were excluded from regression analyses.

**References**

Yang, Z. PAML 4: phylogenetic analysis by maximum likelihood. *Mol. Biol. Evol.* **2007**, *24*, 1586–1591.
