# Supplementary figures and images for: Molecular Evidence for Relaxed Selection on the Enamel Genes of Toothed Whales (Odontoceti) with Degenerative Enamel Phenotypes
Source: Genes (Basel). 2024 Feb 10;15(2):228. doi: 10.3390/genes15020228 (PMC10888366; doi:10.3390/genes15020228)

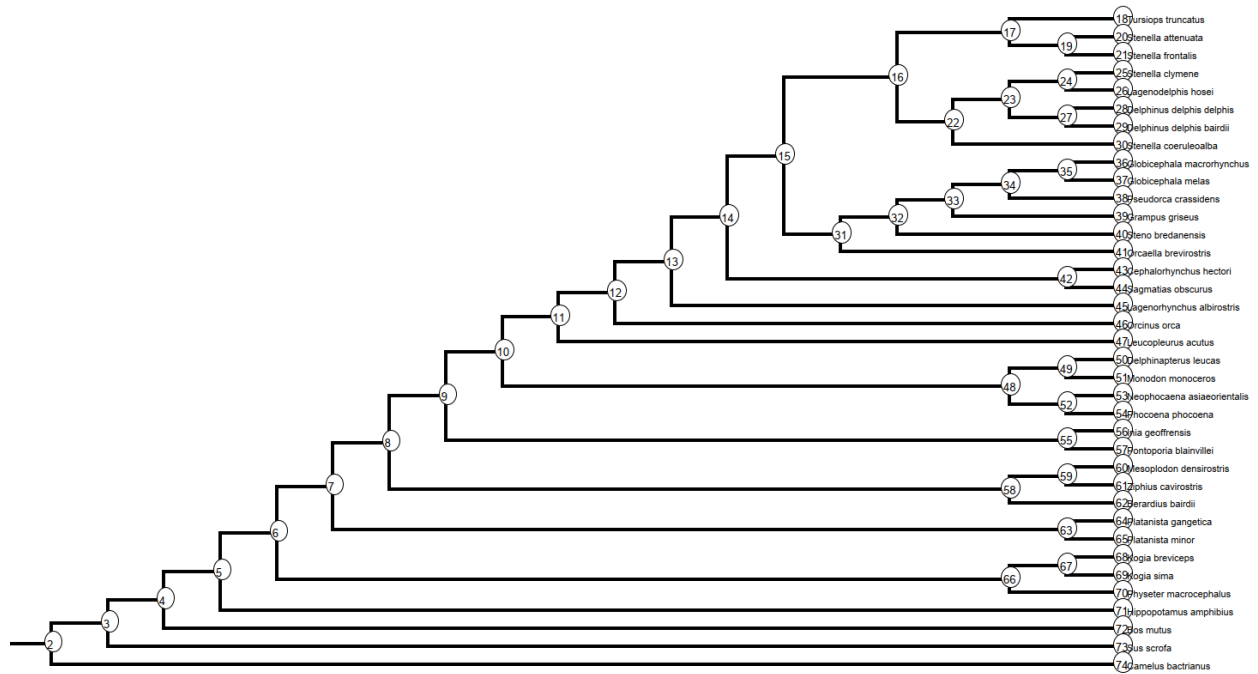

**Figure S1.** Species tree with node numbers for 37 taxa with Werth Enamel Complexity scores.

Supplement: Supplementary file 1 [file genes-15-00228-s001.zip › Supplementary Materials/Supplementary Figures/Figure S1 (37 taxa node numbers).pdf]

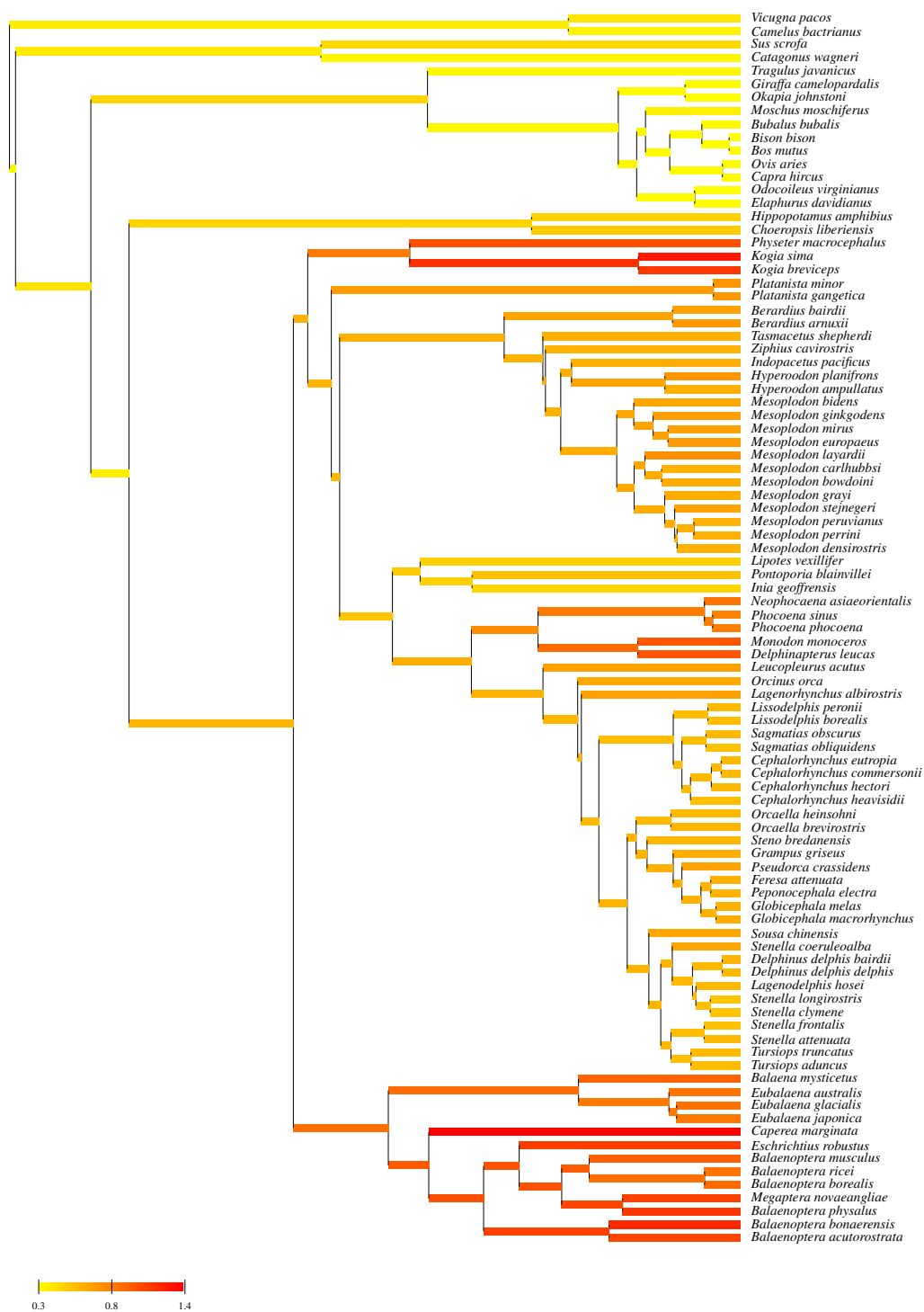

**Figure S8.** Coevol selection analysis (dN/dS) for 93 taxa with species names.

Supplement: Supplementary file 1 [file genes-15-00228-s001.zip › Supplementary Materials/Supplementary Figures/Figure S8 (Coevol species names).pdf]

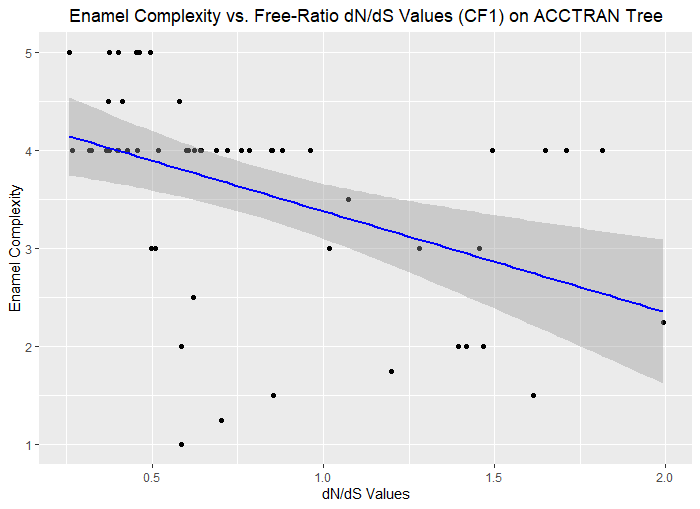

Supplement: Supplementary file 1 [file genes-15-00228-s001.zip › Supplementary Materials/Regression Files/dN:dS/FreeACC1v3.png]

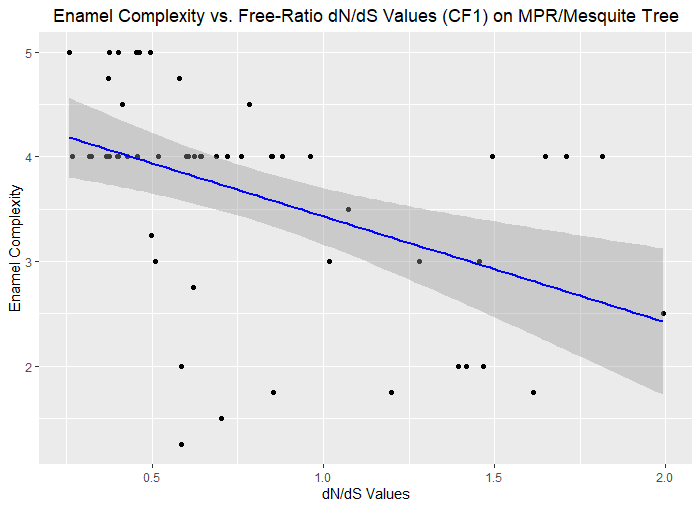

Supplement: Supplementary file 1 [file genes-15-00228-s001.zip › Supplementary Materials/Regression Files/dN:dS/FreeMPR1v3.png]

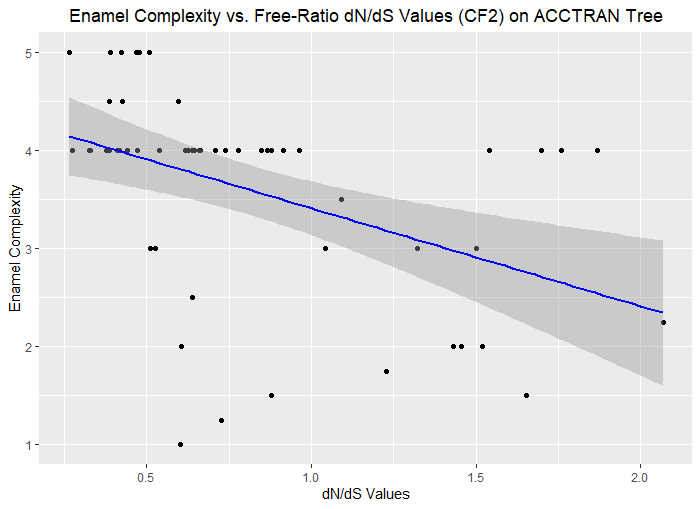

Supplement: Supplementary file 1 [file genes-15-00228-s001.zip › Supplementary Materials/Regression Files/dN:dS/FreeACC2v3.png]

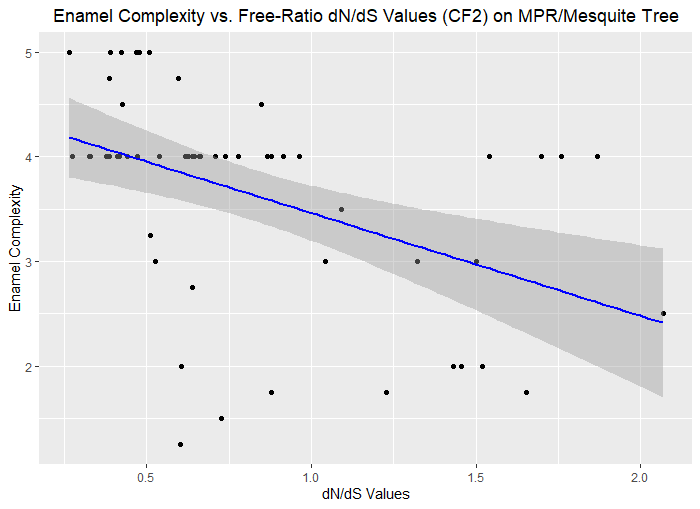

Supplement: Supplementary file 1 [file genes-15-00228-s001.zip › Supplementary Materials/Regression Files/dN:dS/FreeMPR2v3.png]

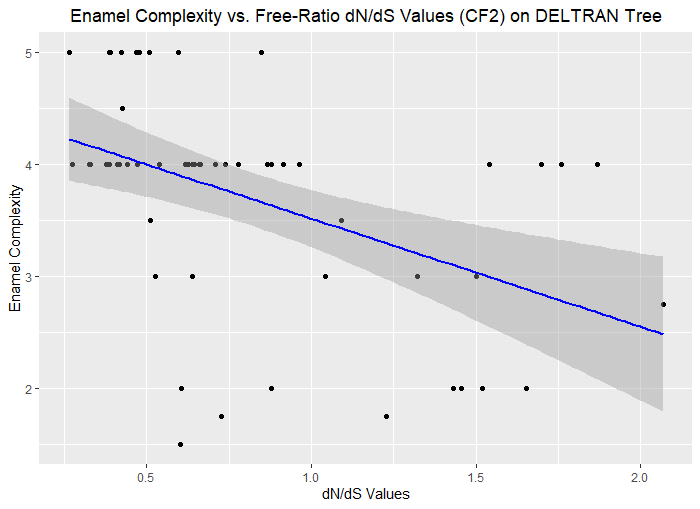

Supplement: Supplementary file 1 [file genes-15-00228-s001.zip › Supplementary Materials/Regression Files/dN:dS/FreeDEL2v3.png]

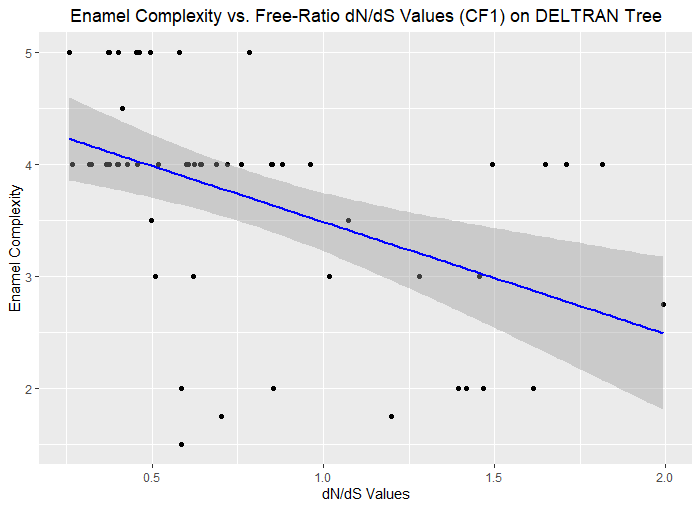

Supplement: Supplementary file 1 [file genes-15-00228-s001.zip › Supplementary Materials/Regression Files/dN:dS/FreeDEL1v3.png]

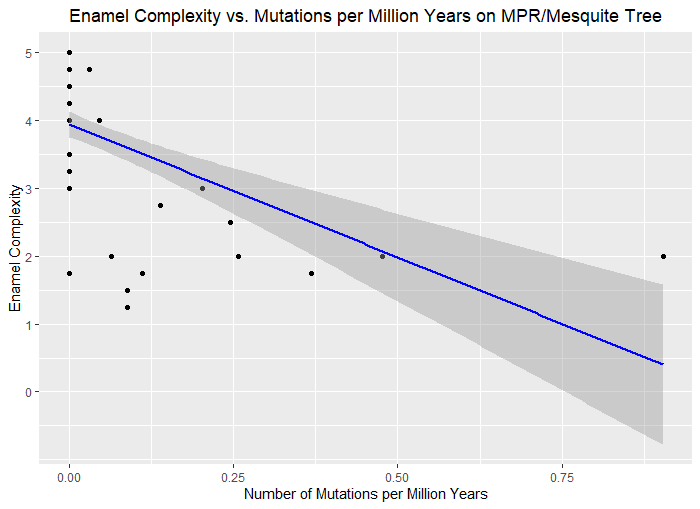

Supplement: Supplementary file 1 [file genes-15-00228-s001.zip › Supplementary Materials/Regression Files/Mutations per Million Years/MutsMYMPRv2.png]

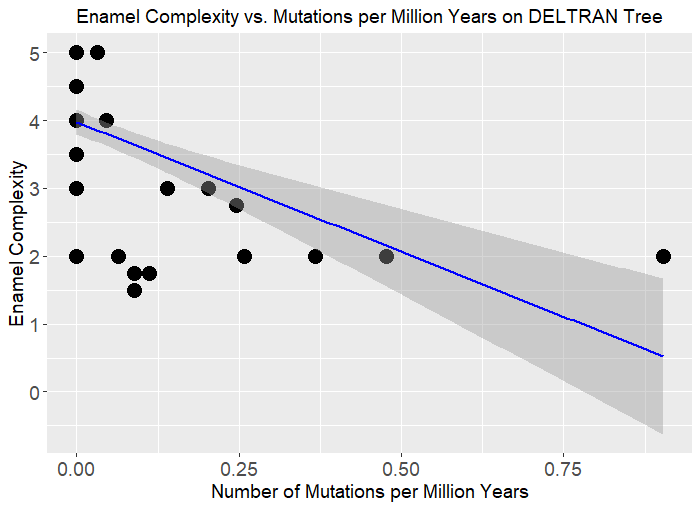

Supplement: Supplementary file 1 [file genes-15-00228-s001.zip › Supplementary Materials/Regression Files/Mutations per Million Years/MutsMYDELv3.png]

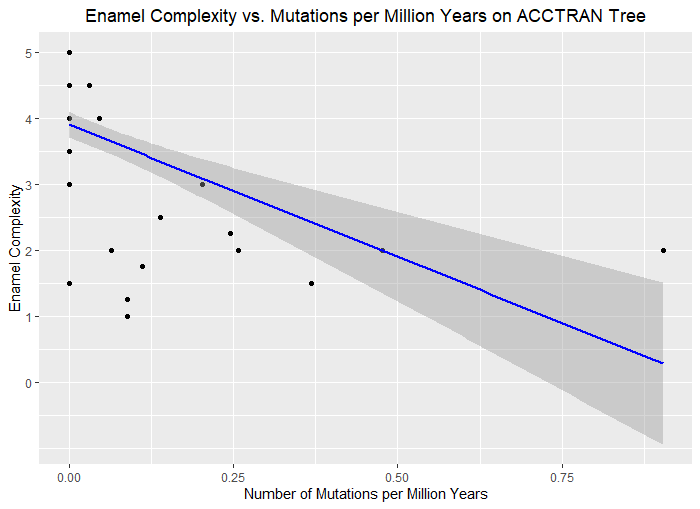

Supplement: Supplementary file 1 [file genes-15-00228-s001.zip › Supplementary Materials/Regression Files/Mutations per Million Years/MutsMYACCv2.png]
